# IL6/adiponectin/HMGB1 feedback loop mediates adipocyte and macrophage crosstalk and M2 polarization after myocardial infarction

**DOI:** 10.3389/fimmu.2024.1368516

**Published:** 2024-03-27

**Authors:** Yue Zheng, Yuchao Wang, Bingcai Qi, Yuheng Lang, Zhibin Zhang, Jie Ma, Minming Lou, Xiaoyu Liang, Yun Chang, Qiang Zhao, Wenqing Gao, Tong Li

**Affiliations:** ^1^ School of Medicine, Nankai University, Tianjin, China; ^2^ Department of Heart Center, The Third Central Hospital of Tianjin, Nankai University Affiliated Third Center Hospital, Tianjin, China; ^3^ Department of Heart Center, Tianjin Key Laboratory of Extracorporeal Life Support for Critical Diseases, Tianjin, China; ^4^ The Third Central Clinical College of Tianjin Medical University, Tianjin, China; ^5^ Department of Heart Center, Tianjin Extracorporeal Membrane Oxygenation (ECMO) Treatment and Training Base, Tianjin, China; ^6^ Department of Heart Center, Tianjin Kang Ting Biological Engineering Group CO. LTD, Tianjin, China; ^7^ State Key Laboratory of Medicinal Chemical Biology, Key Laboratory of Bioactive Materials (Ministry of Education), Frontiers Science Center for Cell Responses, College of Life Sciences, Nankai University, Tianjin, China

**Keywords:** myocardial infarction, adiponectin, macrophage, IL6, lymph-angiogenesis, LNP-MertK

## Abstract

**Background:**

Differences in border zone contribute to different outcomes post-infarction, such as left ventricular aneurysm (LVA) and myocardial infarction (MI). LVA usually forms within 24 h of the onset of MI and may cause heart rupture; however, LVA surgery is best performed 3 months after MI. Few studies have investigated the LVA model, the differences in border zones between LVA and MI, and the mechanism in the border zone.

**Methods:**

The LVA, MI, and SHAM mouse models were used. Echocardiography, Masson’s trichrome staining, and immunofluorescence staining were performed, and RNA sequencing of the border zone was conducted. The adipocyte-conditioned medium-treated hypoxic macrophage cell line and LVA and MI mouse models were employed to determine the effects of the hub gene, adiponectin (*ADPN*), on macrophages. Quantitative polymerase chain reaction (qPCR), Western blot analysis, transmission electron microscopy, and chromatin immunoprecipitation (ChIP) assays were conducted to elucidate the mechanism in the border zone. Human subepicardial adipose tissue and blood samples were collected to validate the effects of ADPN.

**Results:**

A novel, simple, consistent, and low-cost LVA mouse model was constructed. LVA caused a greater reduction in contractile functions than MI owing to reduced wall thickness and edema in the border zone. ADPN impeded cardiac edema and promoted lymphangiogenesis by increasing macrophage infiltration post-infarction. Adipocyte-derived ADPN promoted M2 polarization and sustained mitochondrial quality via the ADPN/AdipoR2/HMGB1 axis. Mechanistically, ADPN impeded macrophage HMGB1 inflammation and decreased interleukin-6 (IL6) and HMGB1 secretion. The secretion of IL6 and HMGB1 increased ADPN expression via STAT3 and the co-transcription factor, YAP, in adipocytes. Based on ChIP and Dual-Glo luciferase experiments, STAT3 promoted ADPN transcription by binding to its promoter in adipocytes. *In vivo*, ADPN promoted lymphangiogenesis and decreased myocardial injury after MI. These phenotypes were rescued by macrophage depletion or HMGB1 knockdown in macrophages. Supplying adipocytes overexpressing STAT3 decreased collagen disposition, increased lymphangiogenesis, and impaired myocardial injury. However, these effects were rescued after HMGB1 knockdown in macrophages. Overall, the IL6/ADPN/HMGB1 axis was validated using human subepicardial tissue and blood samples. This axis could serve as an independent factor in overweight MI patients who need coronary artery bypass grafting (CABG) treatment.

**Conclusion:**

The IL6/ADPN/HMGB1 loop between adipocytes and macrophages in the border zone contributes to different clinical outcomes post-infarction. Thus, targeting the IL6/ADPN/HMGB1 loop may be a novel therapeutic approach for cardiac lymphatic regulation and reduction of cell senescence post-infarction.

## 1 Introduction

Coronary artery disease (CAD) is currently responsible for one in every seven all-cause-related deaths (1). Myocardial injury induced by myocardial infarction (MI) or ischemia/reperfusion (I/R), including percutaneous coronary intervention (PCI), can result in a lower left ventricular ejection fraction (LVEF) and induce life-threatening symptoms, such as severe thrombosis, malignant arrhythmia, and cardiac rupture (2, 3).

Left ventricular aneurysm (LVA), one of the most severe MI complications, usually results in a significantly lower LVEF and can induce life-threatening symptoms, such as malignant arrhythmia and cardiac rupture (4). If a paradoxical movement occurs in an apical aneurysm, heart functions, including LVEF and arrhythmia, are exacerbated (5). Acute ventricular aneurysms usually form within 24 h after the onset of MI and may induce heart rupture; however, LVA surgery is best performed 3 months after MI because the incidence of surgical death is higher within 3 months and waiting enables improved ventricular wall myocardial function and scar formation of the infarcted myocardium (4, 5). Border zone differences contribute to different outcomes post-infarction owing to the stretch force resulting from unstable hemodynamic homeostasis, such as LVA and MI (5). Few studies have focused on the effects of border zones on LVA formation and MI progression. Therefore, the LVA model and efficient treatments to prevent LVA formation should be urgently evaluated.

Adipose tissue regulates granulopoiesis and cardiac functions post-infarction (6, 7). The adipokine, adiponectin, inhibits LPS-induced HMGB1 release in RAW264.7 macrophages (8) and regulates FXR agonism-mediated cardioprotection against post-infarction remodeling and dysfunction (9). Re-activation of the epicardium resulted in cardiac remodeling after myocardial injury via paracrine secretion. Adipose tissues in the epicardium and sub-epicardium also increased *de novo* vessel formation in the peri-infarct zone near the epicardium or the “epicardial border zone,” which may be a novel therapeutic approach for cardiac lymphatic regulation (10, 11).

In the present study, we constructed a simple, consistent, and low-cost LVA mouse model. Differences in the border zone between the MI and LVA groups were investigated, and RNA sequencing of the border zone from the LVA, MI, and SHAM groups was performed. Finally, the effects of the hub gene, adiponectin (ADPN), were explored in cell lines and mouse models.

## 2 Methods

### 2.1 Cell source and processing

The HL-1, 3T3-L1, and RAW264.7 cell lines were purchased from the Chinese Academy of Medical Sciences and were cultured in Dulbecco’s modified Eagle’s medium (DMEM) containing 10% (v/v) fetal bovine serum (FBS) in a 37°C, 5% CO_2_ incubator before the experiment.

Adipocyte differentiation was induced as previously described (12). The cells were cultured in 6-well plates (5×10^5^ cells per well) and transfected. The expression of ADPN or STAT3 in adipocytes was knocked down using 9 μL of lipo2000 (11668-019, Invitrogen), 50 nM of the appropriate siRNA 3 μL [siADPN pool (mmu-ADPN-1, mmu-ADPN-2, and mmu-ADPN-3; RIBIBIO, China) or siSTAT3 pool (mmu-STAT3-1, mmu-STAT3-2, and mmu-STAT3-3; RIBIBIO, China)], and 250 μL of OptiMEM (31985-070, Gibco) per well. STAT3 was overexpressed in adipocytes using lipo2000, STAT3 plasmid (RIBIBIO, China), and OptiMEM. Non-sense sequences were used as negative controls (210011, Ubigene).

RAW 264.7 macrophages with HMGB1 knockdown were obtained using adenovirus (m-HMGB1-shRNA-GFP-Puro from 293T cells, 6.57 × 10^8^ TU/mL, MOI:100, 7.6 L, Genechem, Shanghai). Cells were cultured in 6-well plates (5 × 10^5^ cells per well), transfected using shRNA HMBG1 (MOI = 10), and screened using puromycin (2 μg/mL, Genechem, Shanghai) to obtain the infected RAW 264.7 macrophages.

RAW 264.7 macrophages were maintained in high-glucose DMEM (Gibco, USA) containing 10% fetal bovine serum (AusgeneX, Australia) and 1% penicillin/streptomycin (Solarbio, China) at 37°C under normal (5% CO_2_ and 95% air) or hypoxia (5% CO_2_, 94% N_2_, and 1% O_2_) conditions. To determine the effects of adipocyte-derived ADPN on macrophages, the cells were cultured in the conditioned medium (CM) from siNC or siADPN adipocytes. To determine the macrophage receptor that binds adipocyte-derived ADPN, macrophage receptor-binding antibodies, including AdipoR1 (ab126611), AdipoR2 (ab77612), aVb3 (EMD Millipore, MAB1876-Z), and aVb5 (EMD Millipore, MAB1961), were added to siNC CM, which was then administered to macrophages.

### 2.2 Animals

Adult C57Bl/6J male mice (young: 7–8 weeks old, weight 22–25 g; aged: 20–22 weeks old, weight approximately 35 g) were purchased from Charles River (Beijing, China). Adult experimental Axin2 knockout (KO) mice (background: C57Bl/6J) were fed and propagated at Nankai University. PCR genotyping of Axin2 KO and WT mice was performed using primers 5′-AGTCCATCTTCATTCCGCCTAGC-3′ and 5′-TGGTAATGCTGCAGTGGCTTG-3′ for the wild type, and primers 5′-AGTCCATCTTCATTCCGCCTAGC-3′ and 5′-AAGCTGCGTCGGATACTTGCGA-3′ for the Axin2 mutant.

Mice were maintained in a specific pathogen-free environment with free access to food and water and a 12 h/12 h light–dark cycle. The study protocol was approved by the Ethics Committee of Nankai University (approval no. 2022-SYDWLL-000486).

### 2.3 Mouse model and treatment

Mice were anesthetized via inhalation of isoflurane (1.5%–2%, MSS-3, England). LVA and MI mouse models were established by ligating the left anterior descending (LAD) artery. MI was induced in adult male mice according to previous studies (10), whereas LVA was induced in adult male mice according to previous studies, with a slight modification. Briefly, the heart in the LVA group was ligated at the LAD at the same level as the heart in the MI group; however, the suture was snipped day 5 after the operation. The LVA model was considered to be successfully established based on the paradoxical movement of the left ventricle. The sham-operated animals underwent the same procedure as the MI and LVA model animals but did not undergo coronary artery ligation.

To determine the effects of ADPN on the formation and progression of LVA, ADPN [ip., 5 ng/(g·day), bs0471P, Bioss] was administered from day 1 to day 14, day 1 to day 3, day 1 to day 7, day 3 to day 14, and day 7 to day 14 after LAD ligation. To elucidate the effects of ADPN and HMGB1 on the crosstalk between adipocytes and macrophages, ADPN, clodronate liposomes, and AAV-shHMGB1 were applied in MI and LVA mouse models. Clodronate liposomes (iv., 0.1 mL/25 g, Yeasen) were used to eliminate bone marrow-derived macrophages. AAV-shHMGB1 was administered as a pre-treatment day 14 before the operation to impede HMGB1 expression in macrophages. To determine the effects of the STAT3/ADPN/HMGB1 axis on the crosstalk between adipocytes and macrophages, adipocytes overexpressing STAT3, siSTAT3 adipocytes, and AAV-shHMGB1 were used in the MI and LVA mouse models.

To reduce pain in the animal experiments, the animals were killed via cervical dislocation after isoflurane anesthesia (5%, MSS-3, England).

### 2.4 LVA model validation

To validate the successful construction of the LVA model, hearts were collected on day 28 after the operation, and a regular agarose (agarose G-10, Blowest, 162135) intra-chamber cast was used to recapture the inner chamber spatial structure and the bulging shape of the LVA. To screen the time window of LVA model formation, computed tomography (CT, Nanoscan, Mediso, width: approximately 1,929, center: approximately −35) was applied to perform *in vivo* imaging of mouse cardiopulmonary parts and validate the success of LVA model development at different time points (days 1, 3, 7, and 30 after model construction). Based on paradoxical movement, intrachamber casts, and CT, the LVA mouse model was validated.

### 2.5 Echocardiographic examination

Cardiac function was evaluated using a Vevo^®^ 2100 system equipped with a 30-MHz transducer (FUJIFILM VisualSonics, Inc. Toronto, Canada). Heart function was measured using the two-dimensional parasternal long axis. Left ventricular internal dimensions in diastole (LVID,d), systole (LVID,s), left ventricular ejection fraction (LVEF, %), and fractional shortening (LVFS, %) were measured using M-mode.

### 2.6 Histology analysis

After mice were killed, heart samples were collected, fixed in 4% paraformaldehyde overnight, and embedded in paraffin or OCT (6 μm). Hematoxylin and eosin (HE) staining (G1120, Solarbio, China) and Masson’s staining (G1340, Solarbio, China) were performed to detect anatomical morphology changes and collagen deposition. The average infarct size was obtained by calculating the average length of the circumference in the infarct and normal areas. The infarcted area was determined by calculating the average area in the infarct portion and total area (13). The related wall thickness of the border zone was also determined, and the three were measured using the ImageJ software.

For immunofluorescent analysis, the tissue slides were incubated with the following primary antibodies: rabbit anti-Cx43 (1:200, ab11370), anti-LYVE1 (1:200, ab218535), anti-ZO-1 (1:200, ab61357), mouse anti-CD68 (1:200, ab31630), rabbit anti-HMGB1 (1:200, ab79823), mouse anti-ADPN (1:200, ab22554), mouse anti-cTNT (1:200, ab8295), and rabbit anti-Ki67 (1:200, ab16667) at 4°C overnight, followed by the secondary antibodies, Alexa Fluor 488- and Alexa Fluor 594-conjugated second antibody (Abcam, 1:200). The nuclei were stained with 4′,6-diamidino-2-phenylindole (DAPI; Southern Biotech, USA). At least three different heart sections were obtained from each mouse and more than six random images of the at-risk areas were obtained from each section. Images were captured using a fluorescence microscope (ECLIPSE TS2R, Nikon, Japan).

### 2.7 Cytokine and chemokine measurements

For the protein chip, blood samples were collected and centrifuged for 10 min at 3,000 rpm. The concentrations of cytokine and chemokine were measured using an antibody-coated microsphere-based multiplex cytokine immunoassay that can quantify seven cytokines contemporaneously using 25 μL of mouse serum (MTH17MAG-47k MILLIPLEX MAP Mouse Cytokine/Chemokine Magnetic Bead Panel, MERCK Millipore Corporation, Germany). The following cytokines were measured: GM-CSF, IFN-γ, IL-1β, IL-6, IL-10, IL-22, and TNF-α. All samples, standards, and quality controls were assayed according to the manufacturers’ instructions. The samples were incubated overnight at 4°C and washed using a magnetic plate washer. The plates were read using a multiplex plate reader and its accompanying software (Luminex x-MAP Technology, MILLIPLEX). All cytokine and chemokine concentrations are expressed in pg/mL.

To investigate myocardial injury on days 3 and 7 after construction of the mouse model, the left ventricular heart samples from different groups were collected and the mouse ADPN ELISA kit (ab226900), mouse CKMB ELISA kit (JL12422, Jianglaibio, China), and LDH ELISA kit (JL20162, Jianglaibio, China) were used to measure the levels of the corresponding proteins. The concentrations of cytokines secreted by macrophages were measured using the mouse IL6 ELISA kit (ab222503), mouse HMGB1 ELISA kit (JL13702, Jianglaibio, China), and mouse C1qTNF9 ELISA kit (CSB-E16713h, Cusabio, China). The human IL6 ELISA kit (CSB-E04638h, Cusabio, China), human HMGB1 ELISA kit (CSB-E08223h, Cusabio, China), and human ADPN ELISA kit (CSB-E07270h, Cusabio, China) were utilized to determine protein expression in sub-epicardial adipose tissues. Optical density (OD) was measured using a microplate reader (VICTOR Nivo Multimode Microplate Reader, PerkinElmer). All cytokine concentrations are expressed in pg/mL.

### 2.8 RNA-sequencing of the border zone area

The border zones of the LVA and MI heart samples, and the same area of heart samples from the SHAM group were dissected (*n* = 5 per group). Total RNA was extracted using a TRIzol reagent kit (Invitrogen, Carlsbad, CA, USA). RNA quality was assessed using an Agilent 2100 Bioanalyzer (Agilent Technologies, Palo Alto, CA, USA) and checked via RNase-free agarose gel electrophoresis. After total RNA extraction, eukaryotic mRNA was enriched using oligo (dT) beads, whereas prokaryotic mRNA was enriched by removing rRNA using the Ribo-Zero™ Magnetic Kit (Epicenter, Madison, WI, USA). The enriched mRNA was fragmented into short fragments using fragmentation buffer, reverse transcribed into cDNA with random primers, purified using the QiaQuick PCR extraction kit (Qiagen, Venlo, The Netherlands), end-repaired, extended with a poly(A) tail, and ligated to Illumina sequencing adapters. The ligation products were size-selected by agarose gel electrophoresis, amplified via PCR, and sequenced using Illumina HiSeq2500 (Gene Denovo Biotechnology Co., Guangzhou, China).

The differentially expressed genes (DEGs) were assessed using the “limma” package. Genes with log2|fold change|>1 and *p* < 0.05 were considered DEGs. GO/KEGG pathway analyses were performed using the “clusterProfiler” package in R (14, 15). Statistical significance was set at *p* < 0.05 for the enrichment analyses.

To investigate the hub genes, DEGs between the MI and LVA groups were uploaded to STRING (version 11) to construct a protein–protein interaction (PPI) network (16). The results of STRING analysis were imported into Cytoscape v.3.7.1, and hub genes were investigated using the Cytoscape plug-in, MCODE (17). A significant PPI was identified by a combined score >0.4.

Single-cell sequencing data for hub DEGs were obtained from the Single Cell Portal (https://singlecell.broadinstitute.org/). Single-cell sequencing data of the human heart from a single-cell portal were used to explore the expression of ADPN, AdipoR1, and AdipoR2 (SCP498) (18).

### 2.9 qPCR analysis

The mRNA of heart samples in the border zone or cells was extracted, and qRT-PCR was performed. Total RNA was collected using TRIzol and reverse transcribed. The amplified cDNA samples were then mixed using TB Green^®^ Premix Ex Taq™ II (TaKaRa, RR820). B-actin was used as an internal reference; the 2^−ΔΔCt^ method was used for the calculation. The primer details are listed in **Supplementary Table 1**.

### 2.10 Flow cytometry

To determine the effects of different treatments on macrophage M2 polarization, samples were blocked with anti-Rh Fc receptor binding (Invitrogen, 2296579); stained with CD206 (CD206-FITC, Invitrogen 141704) and Edu (Edu-AF594, Invitrogen 2420611) (cell sample), CD206 and CD86 (CD86-PE/Cyanine7, Invitrogen, 105014) (heart sample), or CD206 and F4/80 (F4/80-PE, Invitrogen 157304) (heart sample); and evaluated using a flow cytometer (Cytometer, USA). Following staining for MertK (MertK-PE/Cyanine7, Invitrogen, 2555774), flow cytometry was performed to determine the effect of LNP-MertK on macrophages. Nystatin, a decreasing endocytic reagent, was used to reverse the above effects (MCE 1400-61-9). Data were analyzed using FlowJo 10.6.2 (BD Biosciences).

### 2.11 Western blot

Proteins were extracted using RIPA buffer (CWBIO, China). The protein concentration was measured using a BCA assay kit. The samples were mixed with SDS-PAGE loading buffer, electrophoresed via SDS-PAGE, and transferred onto Millipore or GE PVDF membranes. The PVDF membranes were blocked with 5% skim milk for 1 h and incubated with primary followed by secondary antibodies (all 1:5,000, Affinity, China). The membranes were then bound to ECL and analyzed using an image system software (Protein Simple, USA).

The following primary antibodies were employed: anti-p65 (1:1,000, CST #6959), anti-p-p65 (1:1,000, ab194726), anti-LC3 (1:2,000, ab192890), anti-ADPN (1:1,000, ab22554), anti-HMGB1 (1:1,000, ab79823), anti-GPX4 (1:1,000, ab125066), anti-P62 (1:1,000, ab109012), anti-YAP (1:1,000, CST#8418S), anti-STAT3 (1:1,000, ab109085), and anti-β-actin (1:5,000, Sigma).

### 2.12 Transmission electron microscope

A transmission electron microscope (TEM) was used to examine the ultrastructure of macrophages. Briefly, cells and heart samples were fixed with glutaraldehyde and osmic acid (Solarbio, China), and then cut into ultrathin sections with a thickness of 50–70 nm using an ultrathin microtome. Ultrathin sections were stained with 2% uranyl acetate and lead citrate. The mitochondria and lysosomes were observed using a TEM (HT7800, HITACHI).

### 2.13 Oxidative stress levels

Raw264.7 cells were cultured under normal oxygen or hypoxic conditions for 8 h. Oxidative stress levels were measured by detecting reactive oxygen species (ROS; S0033; Beyotime, China), according to the manufacturer’s instructions. The fluorescence signal was recorded using 520- and 590-nm lasers and detected using a fluorescence microscope (ECLIPSE TS2R; Nikon, Japan).

### 2.14 Autophagy measurement

Raw264.7 cells were cultured in normal oxygen or hypoxic conditions for 8 h with or without treatment. Autophagy was determined using the MDC method (C3018S, Beyotime, China). The fluorescence signal was recorded using a 355-nm laser and detected using a fluorescence microscope (ECLIPSE TS2R, Nikon, Japan).

### 2.15 Oil O staining

To analyze lipid deposition in adipocytes, Oil O staining (G1262, Solarbio, China) was performed for the different groups. To analyze lipid deposition in the human subepicardial layer, modified Oil O staining (G1261, Solarbio, China) was performed. Images were captured using a light microscope (ECLIPSE E100; Nikon, Tokyo, Japan).

### 2.16 Chromatin immunoprecipitation assay

Macrophages or adipocytes were fixed with formaldehyde at room temperature for 10 min, washed with PBS, and collected. The cells were then placed in an ultrasonic water bath (60 W, SCIENTZ-48, SCIENTZ, China). The long-stranded DNA of the gene was broken into 200- to 1,000-bp DNA fragments and centrifuged at 16,000 × *g* for 15 min at room temperature to obtain the supernatant. A total of 20 µL of supernatant was employed as the input. The c-Jun (ab32137, 2.5-5 µg/10^6^ cells) and STAT3 (ab76315, 2.5-5 µg/10^6^ cells) antibodies and their corresponding IgG antibodies (A7016; Beyotime, China) were added to the remaining supernatant, which was incubated overnight at 4°C. Magnetic beads were then added for 2 h at room temperature (CST #9005). After centrifugation at 16,000 × *g* for 2 min at 4°C, the precipitate was eluted stepwise with a low-salt buffer solution, high-salt buffer solution, and NaCl solution to remove chromatin. EDTA, Tris-HCl, and protease were added to the sample, which was then incubated at 65°C for 1 h. Finally, the phenol-chloroform method was used to extract the 50-µL purified product for PCR detection. The primer details are outlined in **Supplementary Table 2**.

### 2.17 Dual-Glo luciferase experiment

The ADPN promoter [bp −1k/+120 relative to the transcriptional start site (TSS)] was cloned into the luciferase reporter gene vector, pGL3-Basic (Promega). Four 5′-truncated sequences of the ADPN promoter in the luciferase reporter gene vector were transfected into adipocytes from different treatment groups. The Dual-Glo luciferase experiment was carried out 48 h after transfection following the protocol provided by the manufacturer. The OD values were measured using a microplate reader (VICTOR Nivo Multimode Microplate Reader, PerkinElmer). The intensity was calculated by dividing the luciferase value by the renilla value (Promega E2920).

### 2.18 LNP and LNP-MertK construction

Lipid nanoparticle (LNP) and LNP containing MertK (LNP-MertK) were constructed as previously described (19). LNP was administered via the iv route. Of note, LNP can specifically bind to bone marrow-derived macrophages (CD68+). LNP-MertK was used to increase MertK expression and macrophage phagocytosis to eliminate unwanted materials in cardiomyocytes and the microenvironment. Nystatin, which decreases endocytic reagent, was administered via the ip route and used to rescue the effects of LNP-MertK after MI.

### 2.19 Patient enrollment and human sample analysis

To further validate the effects of the IL6/STAT3/ADPN/HMGB1 axis, blood samples were collected from patients after admission, and sub-epicardial samples were collected from patients that underwent coronary artery bypass grafting (CABG). The following inclusion criteria were employed: (1) diagnosed with CAD; (2) normal BMI (BMI ≤ 24 kg/m²), overweight (24 < BMI < 28 kg/m²), or obese (BMI ≥ 28 kg/m²); and (3) patients with complete medical information and laboratory examinations. The following exclusion criteria were employed: (1) history of nephropathy, especially DM; (2) history of hepatopathy; (3) history of diabetic oculopathy; (4) history of tumors; (5) cardiac arrest or ECPR; (6) MODS or irreversible brain damage; (7) surgery within 6 months of this study; (8) aortic insufficiency or aortic dissection; and (9) uncontrolled bleeding. The protocols were approved by Tianjin Third Central Hospital.

Univariate and multivariate logistic regression analyses were performed to assess the correlation between patients with CAD in different BMI groups and the expression levels of ADPN, IL6, and HMGB1. Receiver operating characteristic (ROC) analysis and joint ROC analysis were performed to investigate the diagnostic value of ADPN, IL6, and HMGB1 in patients with CAD in the different BMI groups.

### 2.20 Statistical analysis

Data are presented as mean ± SD, median (Q1–Q3), or frequency (percentage). Statistical analyses were performed using SPSS 23.0. The Shapiro–Wilk normality test, Welch’s *t*-test (two groups), and one- or two-way ANOVA with Bonferroni post-hoc analyses were used for comparisons between different groups. Multiple comparison *p*-values less than 0.05 were considered to indicate statistical significance. Randomization and blinding strategies were used whenever possible.

## 3 Results

### 3.1 Changes in the border zone are critical in LVA formation

The LVA mouse model was successfully constructed and validated using an agarose intrachamber cast and CT (**Supplementary Figure 1**). CT was also performed to screen the time window of LVA formation. Bulging was found to appear before day 7 and then progressively enlarge on day 30. Based on CT, the volume of the bulging left ventricular free wall in the LVA group occupied almost 1/3 of all left ventricles on day 30 after the operation. Furthermore, left ventricular paradoxical movement appeared.

**Figure 1:**
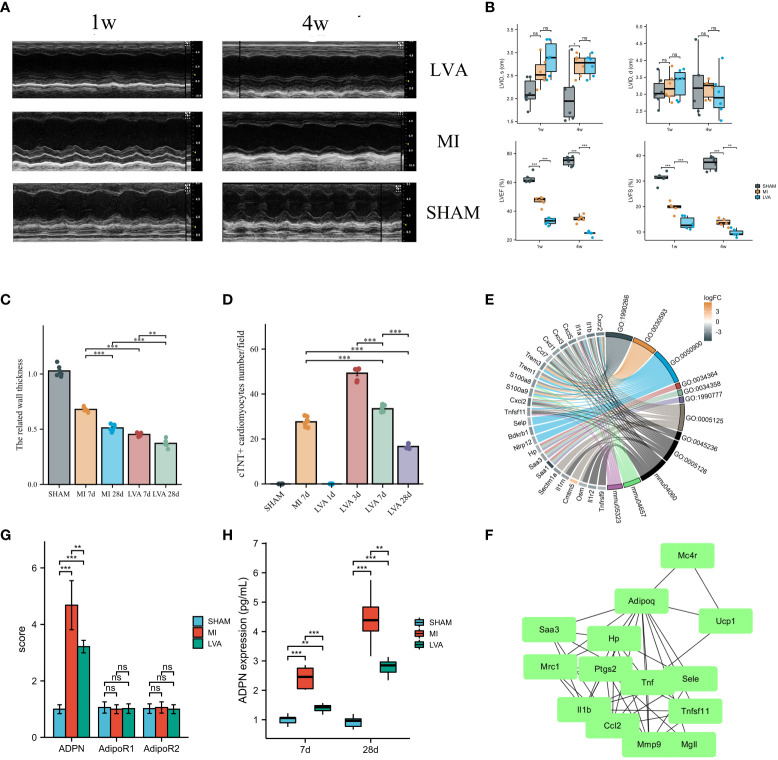
ADPN expression was associated with the wall thickness in the border zone and LVA formation. **(A, B)** LVA and MI decreased heart function. The representative echocardiographic images **(A)** and quantitative analysis **(B)** of LVA, MI and SHAM mice heart samples at 1 week and 4 weeks after the operation. LVID, d, left ventricular internal diameter at end-diastole; LVID, s, left ventricular internal diameter at end-systole; LVEF (%), left ventricle ejection fraction; LVFS (%), left ventricle fractional shortening (*n* = 6 per group). **(C)** The wall thickness in the border zone of SHAM, MI (7 days and 28 days) and LVA (7 days and 28 days) heart samples. **(D)** Immunofluorescent staining of aSMA demonstrated the injury cardiomyocytes in the border zone of SHAM, MI (7 days) and LVA (1 day, 3 days, 7 days, and 28 days) heart samples. **(E)** The chord diagram of the relationship between DEGs and enriched pathways after RNA-sequencing analysis. **(F)** PPI analysis showed the inflammation module of DEGs in the border zone between LVA, MI, and SHAM at 7 days after model construction (*n* = 3 per group). ADPN, adiponectin. **(G)** The qPCR analysis of ADPN, AdipoR1, and AdipoR2 among three groups. AdipoR1 and AdipoR2, the two receptors of ADPN. **(H)** ADPN expression in the border zone at 7 days and 28 days after operation among three groups using ELISA. **p < 0.01; ***p < 0.001; ns, not significant.

Left ventricular functions in the LVA and MI groups were impaired at 1 and 4 weeks after operation compared to those in the SHAM group. LVEF and LVFS in the LVA group were remarkably reduced compared to those in the MI and SHAM groups (**Figures 1A, B**). Masson’s staining and hematoxylin and eosin staining were performed to assess LVA formation (**Supplementary Figures 2A, B**). The infarcted area significantly decreased, while the infarcted size did not significantly change on day 28 compared to that on day 7 in the LVA group (**Supplementary Figures 2D, E**). The wall thickness of the border zone decreased during this process in the LVA group (**Figure 1C**). The infarcted area was significantly larger while the infarct size was significantly lower on day 7 in the LVA group than in the MI group. Immunofluorescent staining for cTNT was observed in the border zone, suggesting that the number of injured cardiomyocytes decreased during LVA formation (**Supplementary Figure 2C**; **Figure 1D**).

Immunoregulation after cardiac injury is critical for heart recovery. Hearts in the LVA group were associated with a “global” activation of pro-inflammatory and anti-inflammatory cytokines (**Supplementary Figure 3**). In the pro-inflammatory phase (<3 days), IL-1b expression was elevated in the LVA and MI groups. Besides the decrease in IL-1β and peak IL-6 at 3 days, other cytokines peaked at 5 days. On day 7 after the operation, the expression of all cytokines was higher in the LVA group than in the MI group, indicating that immunoregulation of the infarcted area and border zone in the LVA heart was more remarkable than that in the MI heart.

Finally, 65 mice were used in the survival study. The mortality rate of LVA mice was higher than that of MI mice. To avoid the influence of the surgical procedure time, we recorded the time but found no significant difference between the two groups (**Supplementary Figure 4**). Taken together, these results indicate that the LVA model was successfully constructed. Many more differences were found in the border zone between the two groups, which may play a critical role in LVA formation and MI progression.

### 3.2 ADPN expression is associated with wall thickness in the border zone in LVA and MI mice

To explore the difference between the LVA and MI border zones, we performed RNA-seq of the border zone in the three groups on day 7 after the operation. A total of 1,340 upregulated and 971 downregulated DEGs were identified between the LVA and MI border zones. Further enrichment analysis was performed, which mainly revealed cytokine–cytokine receptor interactions, granulocyte chemotaxis, and chemokine activity (**Figure 1E**; **Supplementary Table 3**). PPI network analysis was performed, and a module comprising TNF, IL1b, ADPN, UCP1, and CCL2 was obtained (**Figure 1F**). Quantitative polymerase chain reaction (qPCR) analysis and ELISA were performed to determine ADPN expression. The mRNA and protein levels of ADPN were lower in the LVA group than in the MI group. No significant difference in mRNA levels was found between the two ADPN receptors, suggesting that ADPN expression may be associated with the related wall thickness of the border zone and contribute to LVA formation and MI progression (**Figures 1G, H**).

### 3.3 ADPN impedes cardiac edema and promotes cardiac lymphangiogenesis post-infarction

**Figure 2:**
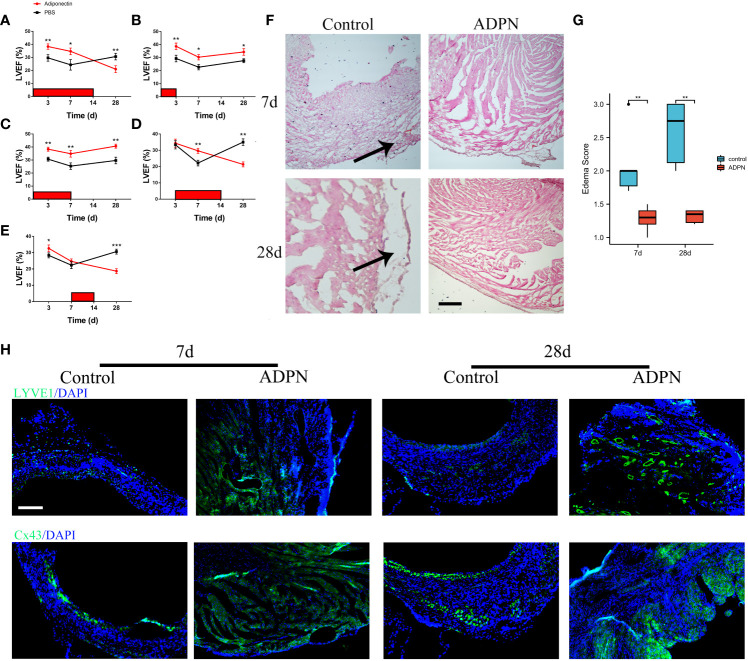
ADPN impeded edema and promoted lymphangiogenesis within 7 days after the operation. **(A–E)** Effect of ADPN treatment on cardiac functions during LVA formation. 5 ng/(g·day) ADPN was injected i.p. from day 1 to 14 **(A)**, day 1 to 3 **(B)**, day 1 to 7 **(C)**, day 3 to 14 **(D)**, and day 7 to 14 **(E)** after LAD ligation. LVEF (%) was evaluated by echocardiography. (*n* = 8 per group) **(F, G)** The representative images **(F)** and quantity analysis **(G)** of the HE staining at 7 days and 28 days in the border zone of the LVA group treated with ADPN or PBS from day 1 to 7 after operation. Scale bar, 500 μm. Arrow, cardiac edema. **(H)** The representative immunofluorescence images of LYVE1 and Cx43 cells in the LVA group treated with ADPN or PBS from day 1 to 7 after operation. Scale bar, 500 μm. *p < 0.05; **p < 0.01; ***p < 0.001.

To explore the effects of ADPN on the related wall thickness of the border zone and LVA formation, an echocardiogram was recorded and the LVEF was measured. Compared to the LVEF in LVA mice administered PBS, the LVEF in mice administered ADPN increased from day 1 to day 7 while that in mice administered ADPN decreased after day 7 (**Figures 2A–E**; **Supplementary Table 4**). When this mechanism was further explored, ADPN supplementation decreased cardiac edema and impeded the progression of subepicardial edema in LVA mice within day 7 after the operation (**Figures 2F, G**). Cardiac lymphatic growth and Cx43 expression in the border zone of ADPN-treated mice were higher than those in age-matched PBS-treated mice on day 28 after the operation (**Figure 2H**).

To determine the effects of ADPN on cardiac lymphangiogenesis and LYVE1+ macrophages in the border zone, ADPN and the Cx43 agonist and inhibitor, verapamil and Gap19, were administered to the LVA mouse model; verapamil and Gap19 were used to regulate Cx43 expression in the border zone. The LVA border zone in the verapamil group exhibited increased Cx43- and Cx43-associated macrophage infiltration (**Supplementary Figure 5A**). LYVE1+ macrophages and lymphatic capillaries increased in the border zone following treatment with ADPN+verapamil compared to treatment with ADPN alone; these levels were restored using Gap19 (**Supplementary Figures 5C, D**).

According to a previous report, ADPN decreases Axin2 expression (20). To explore the effects of ADPN on Cx43 and Cx43-associated macrophage infiltration, Axin2 KO mice were used. The overexpression of Wnt canonical signaling rescued the effects of Cx43 expression and Cx43-associated macrophage infiltration (**Supplementary Figure 5B**). LYVE1+ macrophages and lymphatic capillaries were also rescued in the border zone of Axin2 KO mice (**Supplementary Figures 5E, F**). Co-staining of ZO-1 and Cx43 was higher in the ADPN-treated LVA group than in the non-treated LVA group (**Supplementary Figure 5G**).

Taken together, these results indicate that ADPN expression levels differed after coronary occlusion, leading to a significant difference in the number of lymphangiogenesis and infiltrated LYVE1+ macrophages, inducing different statuses of the border zone and different mouse models, such as MI and LVA (**Supplementary Figure 5H**).

### 3.4 Adipocyte-derived ADPN promotes macrophage M2a polarization and decreases inflammatory cytokine secretion via the ADPN/AdipoR2/HMGB1 axis

**Figure 3:**
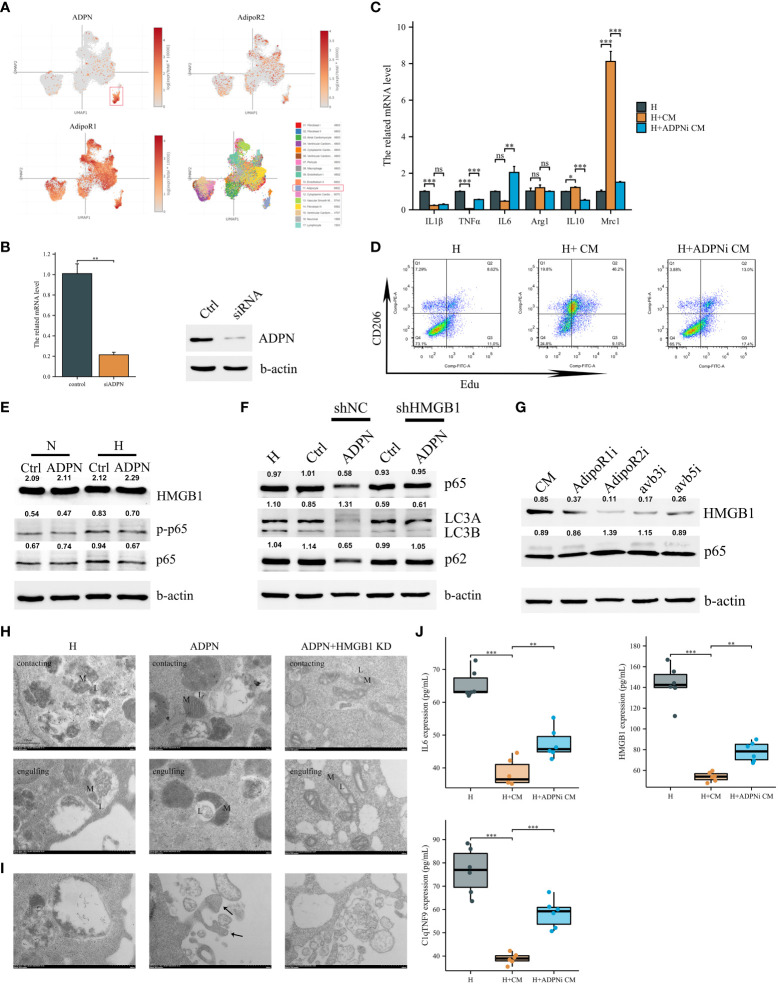
Adipocyte-derived ADPN promoted macrophage M2a polarization and decreased inflammatory cytokine secretion via the ADPN/AdipoR2/HMGB1 axis. **(A)** Single-cell sequencing analysis demonstrated that ADPN was mainly expressed in adipocytes in the subepicardial layer while the two ADPN receptors were expressed in almost cells in heart. **(B)** The ADPN knockdown efficiency in adipocytes using qPCR and WB analysis. **(C)** The polarization-related mRNA levels in hypoxic macrophages were measured using qPCR analysis when treated with the conditional medium of ADPN knockdown or control adipocytes. CM, conditional medium. **(D)** Flow cytometry of CD206 and Edu staining to determine the M2 polarization in hypoxic macrophage when treated with the conditional medium of ADPN knockdown or control adipocytes. **(E)** WB analysis demonstrated HMGB1 and inflammatory response in normoxic and hypoxic macrophages when treated with ADPN or control. **(F)** After HMGB1 knockdown, ADPN effects on inflammatory response and autophagy were rescued in hypoxic macrophages. **(G)** WB analysis showed that HMGB1 and inflammatory response in hypoxic macrophages when treated with the conditional medium of adipocytes and binding with macrophage AdipoR1, AdipoR2, avb3, and avb5 using the antibodies. **(H)** HMGB1 knockdown or control RAW264.7 macrophages were exposed to hypoxia with ADPN treatment, then were fixed by high-pressure freezing and analyzed by TEM. Mitochondria–lysosome contacts (contacting) and megamitochondria engulfing lysosome (engulfing) were shown in different groups. L, lysosome, or lysosome-related organelle; M, mitochondrion. **(I)** The unfit materials ejection from macrophages using TEM. Arrow, migrasomes. Scale bar, 500 nm. **(J)** The IL6, HMGB1, and C1qTNF9 expressions secreted by hypoxic macrophages when treated with the conditional medium of ADPN knockdown or control adipocytes. ADPNi, ADPN knockdown in adipocytes. *p < 0.05; **p < 0.01; ***p < 0.001; ns, not significant.

Single-cell sequencing revealed that ADPN was mainly secreted by adipocytes, and the two receptors were expressed in almost all cardiac cells (**Figure 3A**). Therefore, cell–cell crosstalk may occur after MI. To explore the effects of ADPN on macrophages, ADPN expression was knocked down in adipocytes, and RAW264.7 macrophages were cultured with siNC or siADPN adipocyte-conditioned medium (**Figure 3B**). The siNC adipocyte-conditioned medium promoted macrophage M2 polarization based on polarization-related mRNAs and CD206 and Edu staining via flow cytometry. This effect was decreased by treatment with siADPN adipocyte-conditioned medium (**Figures 3C, D**). HMGB1 expression increased while p-p65 expression decreased in normoxic and hypoxic macrophages treated with ADPN (**Figure 3E**).

Based on previous reports, endogenous HMGB1 mediates ferroptosis and autophagy (21). HMGB1 was found to be knocked down in hypoxic macrophages (**Supplementary Figure 6A**). ROS levels in the hypoxic HMGB1 knockdown macrophage group were markedly higher than those in the hypoxic and normal oxygen macrophage groups (**Supplementary Figure 6B**). Autophagy was increased using the MDC method. Furthermore, LC3B/A expression increased in the hypoxia macrophage group compared to that in the normal oxygen macrophage group. This change was rescued in the hypoxia HMGB1 knockdown macrophage group (**Supplementary Figures 6C, D**). The expression of the ferroptosis marker, GPX4, increased in the hypoxia HMGB1 knockdown macrophage group (**Supplementary Figure 6D**). Macrophage HMGB1 knockdown decreased IL-1β and TNF-α, and increased IL-6, Arg1, IL-10, and Mrc1 expression under the hypoxia condition, indicating macrophage M2a polarization (**Supplementary Figure 6E**).

To determine the effects of adipocyte-derived ADPN on HMGB1 and HMGB1-mediated pathways in macrophages, HMGB1 knockdown or control macrophages were treated with ADPN. HMGB1 knockdown rescued the effects of ADPN on HMGB1-mediated p65 and decreased autophagy (**Figure 3F**). After treatment with CM and the AdipoR2 receptor antibody, HMGB1 expression decreased and p65 expression increased, suggesting that the ADPN/AdipoR2/HMGB1/p65 axis regulates immune responses in macrophages (**Figure 3G**). The effects of ADPN and HMGB1 on macrophage mitochondria were also investigated. Based on the results, ADPN decreased megamitochondria engulfing lysosome and promoted migrasome formation in hypoxic macrophages. The phenotypes were also rescued by HMGB1 knockdown in macrophages (**Figures 3H, I**). siNC adipocyte-conditioned medium impeded IL6, HMGB1, and C1qTNF9 secretion from macrophages, whereas the effect was reversed by treatment with siADPN adipocyte-conditioned medium (**Figure 3J**).

**Figure 4:**
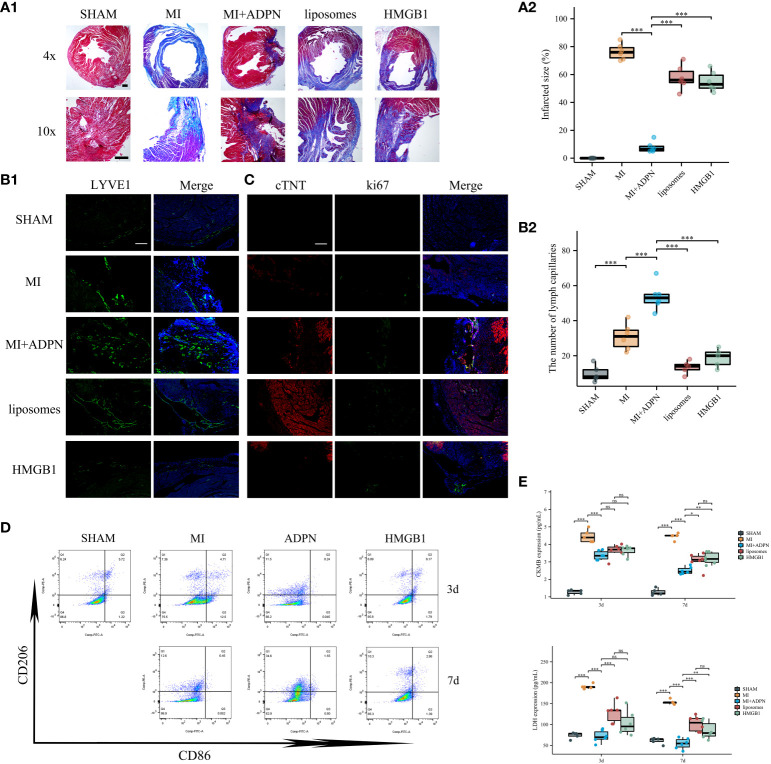
ADPN impeded MI progression via macrophage HMGB1. **(A)** The fibrosis representative images (A1) and quantitative analysis (A2) of heart sections using Masson’s staining in SHAM, MI, MI+ADPN, MI+ADPN+liposomes administration and MI+ADPN+ HMGB1 knockdown in macrophages groups. AAV-shHMGB1 was utilized to knock down HMGB1 in macrophages. **(B)** The immunofluorescent images with LYVE1 (B1) and quantitative analysis (B2) in different groups. **(C)** The immunofluorescent images with Ki67 and cTNT in different groups. cTNT, injured cardiomyocyte marker. **(D)** Flow cytometry of CD206 and CD86 staining to determine the M2a and M2b polarization in hypoxic macrophage in an MI mouse model when treated with ADPN or ADPN+AAV-shHMGB1. **(E)** The border zone CKMB expression (up) and LDH expression (down) at 3 days and 7 days after operation in different groups using ELISA. Liposomes, MI+ADPN+clodronate liposomes administration group; HMGB1, MI+ADPN+HMGB1 knockdown in macrophages. Scale bar, 500 μm. *p < 0.05; **p < 0.01; ***p < 0.001; ns, not significant.

The effects of the ADPN/HMGB1 axis on macrophages and MI progression were validated *in vivo* using an MI mouse model. Compared to the heart sections in the MI group, the fibrotic area decreased after treatment with ADPN; this phenotype was rescued by macrophage depletion (liposome group) or HMGB1 knockdown in macrophages (**Figure 4A**). The same trend was observed for the other effects of ADPN. Treatment with ADPN promoted lymphangiogenesis and increased the number of ki67+ cTNT+ cardiomyocytes after MI. However, these phenotypes were rescued by macrophage depletion or HMGB1 knockdown (**Figures 4B, C**). ADPN was found to promote M2a polarization. After macrophage HMGB1 knockdown, M2a polarization transformed into M2b polarization on days 3 and 7 post-infarction (**Figure 4D**). Treatment with ADPN also decreased LDH and CKMB expression on days 3 and 7 after surgery. These phenotypes were rescued by macrophage depletion or HMGB1 knockdown in macrophages (**Figure 4E**).

Taken together, these results highlight the effects of the ADPN/HMGB1 axis on the regulation of macrophage polarization and cardiac injury in cardiac stress and MI mouse models.

### 3.5 IL6 and HMGB1 promotes ADPN expression in adipocytes via STAT3 and the co-transcription factor, YAP

**Figure 5:**
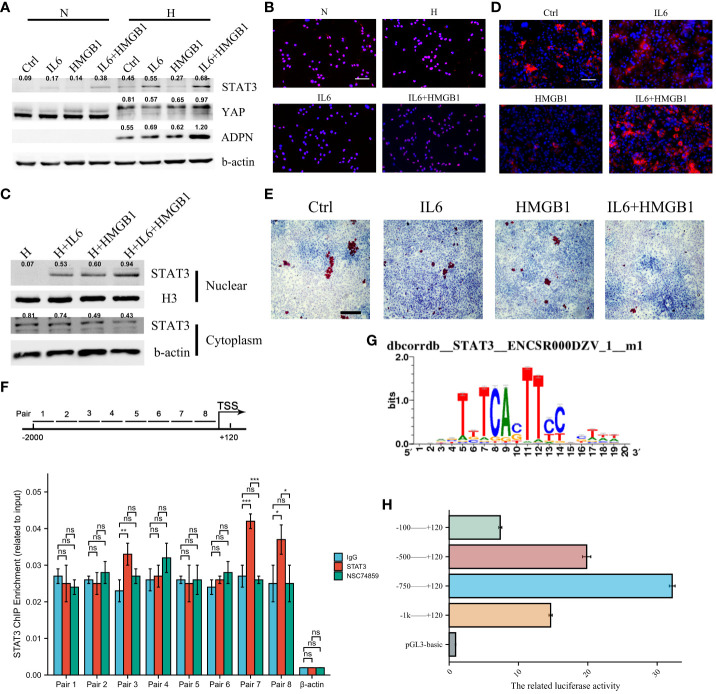
IL6 and HMGB1 promoted ADPN expression in adipocytes via STAT3 and co-transcriptional factor YAP. **(A)** STAT3, YAP, and ADPN expressions were determined in normoxic and hypoxic adipocytes pre-treated with control, IL6, HMGB1, or both. **(B)** Immunofluorescent staining was used to show STAT3 distribution in hypoxic adipocytes pre-treated with control, IL6, HMGB1, or both. Scale bar, 100 μm. **(C)** Nuclear plasma separation experiment demonstrated STAT3 distribution in hypoxic adipocytes pre-treated with control, IL6, HMGB1, or both. **(D)** Immunofluorescent staining with ADPN in hypoxic adipocytes pre-treated with control, IL6, HMGB1, or both. Scale bar, 100 μm. **(E)** Oil O staining was used in hypoxic adipocytes pre-treated with control, IL6, HMGB1, or both. Scale bar, 500 μm. **(F)** ChIP analysis demonstrated that STAT3 promoted ADPN transcription. NSC74859 was used in adipocytes to inhibit STAT3 expression, which was as a negative control. **(G)** The motif of STAT3 and ADPN promoter. **(H)** Luciferase reporter gene experiment was used to validate whether STAT3 promoted ADPN transcription. *p < 0.05; ***p < 0.001; ns, not significant.

We investigated the transcriptional regulation of ADPN in adipocytes. IL6 promoted STAT3 expression in adipocytes, and IL6+HMGB1 promoted a higher expression of STAT3 and YAP in adipocytes (**Figure 5A**). Immunofluorescent staining with STAT3 and nuclear plasma separation experiments revealed that STAT3 translocation to the nucleus increased in hypoxic adipocytes pre-treated with IL6 and markedly increased in adipocytes pre-treated with IL6+HMGB1 (**Figures 5B, C**). ADPN expression was elevated in hypoxic adipocytes pre-treated with IL6 and markedly increased in adipocytes pre-treated with IL6+HMGB1. Lipid deposition was found to decrease in the IL6 and IL6+HMGB1 hypoxic adipocyte groups (**Figures 5D, E**). ChIP analysis and luciferase reporter gene experiments revealed that STAT3 promoted ADPN transcription by binding to the ADPN promoter (**Figures 5F–H**).

**Figure 6:**
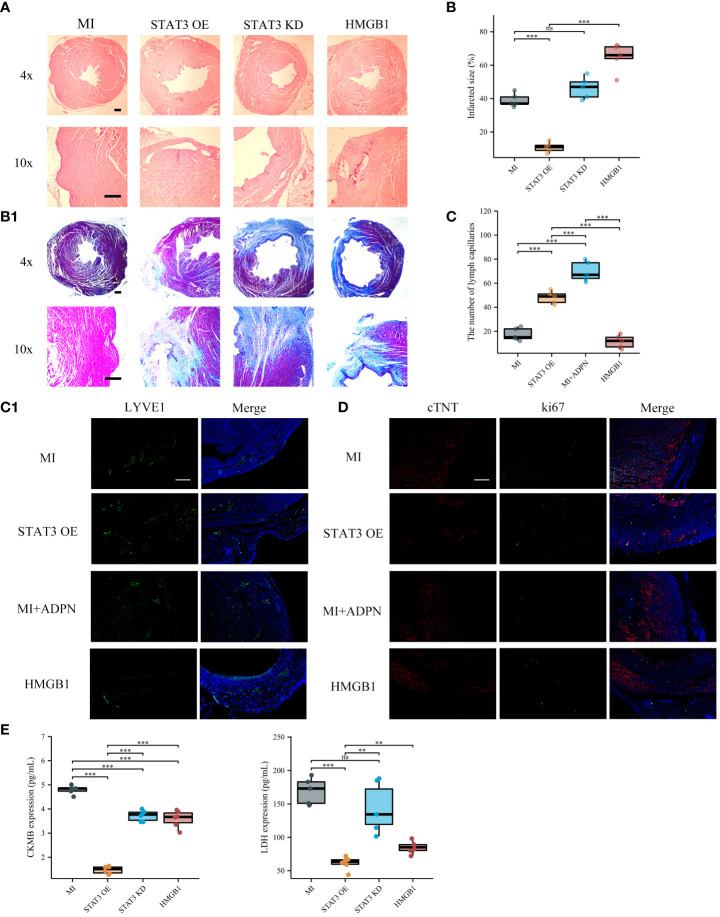
Supplying STAT3 overexpressed adipocytes increased lymphangiogenesis after MI via macrophage HMGB1. **(A)** The HE representative images of heart sections in MI, MI+STAT3 overexpressed adipocytes, MI+STAT3 knockdown adipocytes, and MI+STAT3 overexpressed adipocytes+HMGB1 knockdown macrophage groups. AAV-shHMGB1 was utilized to knock down HMGB1 in macrophages. **(B)** The fibrosis representative images (B1) and quantitative analysis (B2) of heart sections using Masson’s staining in MI, MI+STAT3 overexpressed adipocytes, MI+STAT3 knockdown adipocytes, and MI+STAT3 overexpressed adipocytes+HMGB1 knockdown macrophage groups. **(C)** The immunofluorescent images with LYVE1 (C1) and quantitative analysis (C2) in MI, MI+STAT3 overexpressed adipocytes, MI+ADPN treatment, and MI+STAT3 overexpressed adipocytes+HMGB1 knockdown macrophage groups. MI+ADPN treatment as the positive control. **(D)** The immunofluorescent images with Ki67 and cTNT in MI, MI+STAT3 overexpressed adipocytes, MI+ADPN treatment, and MI+STAT3 overexpressed adipocytes+HMGB1 knockdown macrophage groups. MI+ADPN treatment as the positive control. cTNT, injured cardiomyocyte marker. **(E)** The border zone CKMB expression (up) and LDH expression (down) at 7 days after operation in different groups using ELISA. HMGB1, MI+STAT3 overexpressed adipocytes+HMGB1 knockdown macrophage group. Scale bar, 500 μm. **p < 0.01; ***p < 0.001; ns, not significant.

To validate the effects of the IL6/STAT3/ADPN axis on MI progression and LVA formation, adipocytes overexpressing STAT3, siSTAT3 adipocytes (as a negative control), and AAV-HMGB1 were administered to the MI and LVA mouse models. Supplying adipocytes overexpressing STAT3 decreased collagen disposition, increased lymphangiogenesis, and impaired myocardial injury (LDH and CKMB ELISA results, and immunofluorescent images with Ki67 and cTNT). However, these effects were rescued after HMGB1 knockdown in macrophages (**Figure 6**). Supplying adipocytes overexpressing STAT3 to the LVA mouse model increased the related wall thickness and decreased LDH and CKMB at 7 days after the operation (**Supplementary Figure 7**). Overall, adipocyte-derived STAT3 impedes MI and LVA progression via HMGB1 in macrophages.

### 3.6 Targeting MertK improves heart functions after MI

Due to the aging-related reduction in MertK expression and macrophage phagocytosis, we constructed and used LNP-MertK to increase macrophage MertK expression and phagocytosis. Thereafter, we determined whether LNP-MertK can exhibit a synergistic effect with ADPN on MI progression. First, we analyzed the homing of senescent macrophages to the bone marrow. The homing of senescent macrophages was found to increase in aged mice. These macrophages may decrease the immune response, promote cardiac adverse remodeling, and induce LVA formation (**Supplementary Figure 8A**). Echocardiography revealed that LVEF and LVFS in the LNP-MertK group were higher than those in the MI and LNP groups (**Supplementary Figure 9A**). Masson’s trichrome staining also revealed a smaller infarcted area and more surviving cardiomyocytes in the LNP-MertK group (**Supplementary Figure 9B**). Transwell analysis revealed that the phagocytosis of LNP-MertK-treated macrophages was increased and rescued by nystatin. Macrophage phagocytosis decreased to zero upon treatment with nystatin (**Supplementary Figure 9C**). Based on flow cytometry, LNP-MertK promoted macrophage phagocytosis in the more myocardial adverse mitochondria (**Supplementary Figure 9D**). LNP-MertK also promoted M1 polarization at day 3 and M2 polarization at day 7 after operation (**Supplementary Figures 8E**, **7B**). ADPN enhanced the effects of LNP-MertK on M2 polarization at day 7 after the operation (**Supplementary Figure 8B**). Both LNP-MertK and LNP decreased CKMB and LDH expression; however, LNP-MertK expression decreased further after MI (**Supplementary Figure 9F**). LNP-MertK and ADPN increased the abundance of the LYVE1+ MertK+ macrophages compared with LNP-MertK alone (**Supplementary Figure 9G**). LNP-MertK also increased VEGF-C expression on days 3 and 7 after MI (**Supplementary Figure 9H**). Overall, targeting MertK increased macrophage phagocytosis and M2 polarization, and impeded macrophage senescence, thereby improving heart function after MI.

### 3.7 IL6/ADPN/HMGB1 axis contributes to the diagnosis and prognosis of aging patients with CABG

**Figure 7:**
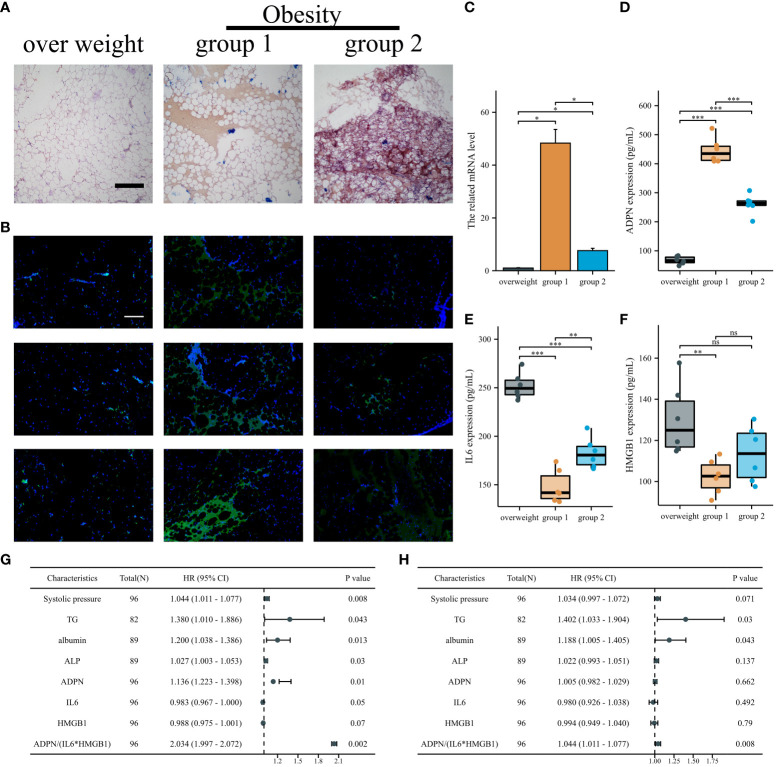
IL6/ADPN/HMGB1 axis contributed to the diagnosis and prognosis of CABG patients. **(A)** Oil O staining demonstrated the lipid deposition of sub-epicardial adipocytes in patients with overweight and obesity. Scale bar, 500 µm. **(B)** Immunofluorescent staining with ADPN in sub-epicardial adipocytes in patients with overweight and obesity. Scale bar, 500 µm. **(C)** The ADPN mRNA level was measured using qPCR in sub-epicardial adipocytes in patients with overweight and obesity. **(D–F)** ADPN **(D)**, IL6 **(E)**, and HMGB1 **(F)** expressions of human sub-epicardial samples in patients with overweight and obesity were determined using ELISA. **(G, H)** Univariate **(G)** and multivariate logistic regression analysis **(H)** of young, middle, and aged CABG patients. *p < 0.05; **p < 0.01; ***p < 0.001; ns, not significant.

To validate the effects of IL6, HMGB1, and ADPN in humans, human subepicardial and blood samples were collected based on the inclusion and exclusion criteria. Patients with obesity and CAD can be divided into two groups based on the lipid deposition of sub-epicardial adipocytes analyzed by oil O staining. Lipid deposition was negatively correlated with ADPN immunofluorescence staining in sub-epicardial adipocytes (**Figures 7A, B**). The high mRNA and protein levels of ADPN were confirmed using qPCR and ELISA (**Figures 7C, D**). However, the levels of secreted IL6 and HMGB1 were found to be downregulated using ELISA (**Figures 7E, F**).

The expression levels of IL6, HMGB1, and ADPN in human blood samples were determined using ELISA. The baseline characteristics of young, middle-aged, and aged patients with CABG are shown in **Supplementary Table 5**. Systolic pressure, albumin, and ADPN were positively correlated with aging in patients with CABG, while IL6 and HMGB1 were negatively correlated with aging (**Supplementary Table 5**). According to univariate and multivariate logistic analyses, TG, albumin, and ADPN/(IL6*HMGB1) are independent factors in young, middle, and aging patients with CABG, highlighting the clinical value of the IL6/ADPN/HMGB1 axis in aging patients with CABG (**Figures 7G, H**; **Supplementary Table 6**).

**Figure 8:**
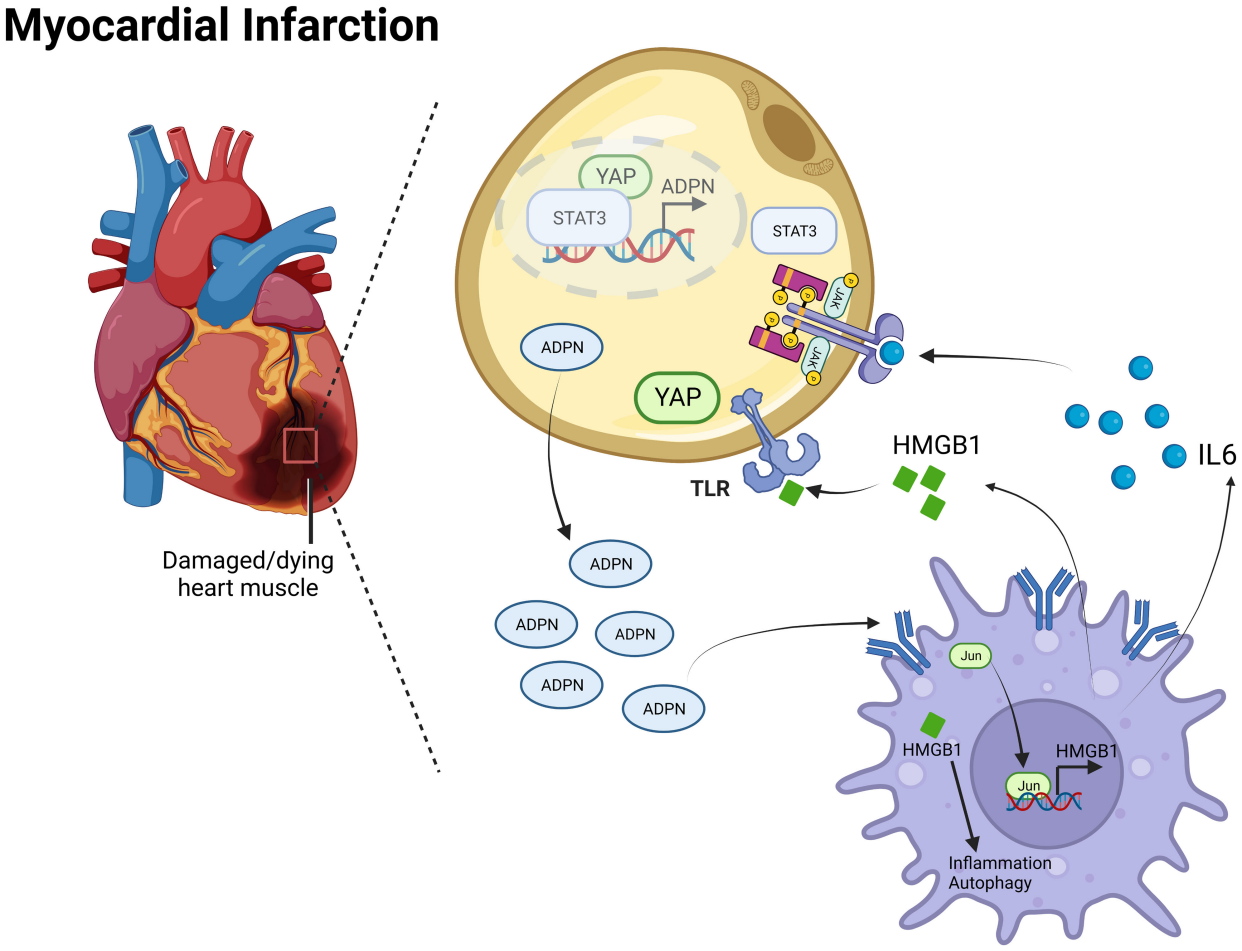
Mechanism diagram of the IL6/ADPN/HMGB1 axis on crosstalk between adipocytes and macrophages after MI. ADPN regulated HMGB1 expression and HMGB1-mediated inflammatory response and autophagy in macrophages via AdipoR2/c-Jun. Moreover, ADPN impeded HMGB1and IL6 secretion in hypoxic macrophages, especially in aged hypoxic macrophages. IL6 triggered STAT3 and HMGB1 promoted YAP translocate to nuclear, which raised ADPN expression through binding to ADPN promoter. Therefore, the IL6/ADPN/HMGB1 axis contributed to macrophage phagocytosis, senescence, inflammatory response, and autophagy, which led to MI progression, LVA formation, and CAD patients’ heterogeneity.

Overall, the IL6/ADPN/HMGB1 axis was found to be differentially expressed in human subepicardial tissue and blood samples. Moreover, the independent factors for aging patients with CABG led to MI progression, LVA formation, and heterogeneity among patients with CAD (**Figure 8**).

## 4 Discussion

LVA is a severe complication of MI. The clinical manifestations of ventricular remodeling include LV expansion and a reduction in cardiac systolic and diastolic functions (22). In this study, we constructed an LVA mouse model to determine the mechanism of LVA formation and the differences in the border zone between LVA and MI hearts. RNA sequencing and further experiments revealed that the hub gene, ADPN, was involved in the crosstalk between adipocytes and macrophages after MI, LVA, and cardiac stress in a mouse model. Thus, ADPN could be a potential target to block LVA formation and MI progression, especially in patients with CAD and obesity or type 2 diabetes.

Cardiac remodeling after MI relies on a balance between debris removal and scar formation (23), and hemodynamic homeostasis during LVA formation. In this study, the stretch force near the plication site increased, inducing the angle of the border zone at 4 weeks after the operation, which may be due to unstable hemodynamic homeostasis, including turbulent flow in the left ventricle. The instable hemodynamic homeostasis led to the related poor stress resistance in the border zone and LVA formation, which was consistent with the results of Jackson et al. (24) and Ratcliffe et al. (25). These investigators found that the stretch force toward the border zone contributed to its extension, called the “diluted” border zone.

The “diluted” border zone was correlated with LVA formation due to the imbalance between border zone stress and left ventricular hemodynamic instability. Cytokine levels were determined to analyze the immunoregulatory mechanism in LVA formation. LVA was suggested to induce more remarkable inflammatory reactions than MI. After RNA sequencing and validation, ADPN was associated with border zone thickness and LVA formation. ADPN impeded cardiac edema and promoted cardiac lymphangiogenesis post-infarction. Trauma induced myocardial ischemia/reperfusion injury by increasing apoptosis, enlarging infarct size, and decreasing cardiac function. Plasma adiponectin concentrations decreased after traumatic injury. Both etanercept and exogenous adiponectin supplementation (3 days post-trauma or 10 min before reperfusion) markedly inhibited oxidative/nitrative stress and ischemia/reperfusion injury in WT mice, whereas adiponectin supplementation substantially attenuated post-traumatic ischemia/reperfusion injury in adiponectin-knockout mice (26). As ADPN has the same domain as TNFa, TNF-α antagonism was found to ameliorate myocardial ischemia-reperfusion injury in mice by upregulating adiponectin (27). The cardioprotective effects of TNF-α neutralization are partially due to the upregulation of ADPN based on a comparison of ADPN knockout mice with WT mice (26, 27).

The resolution of cardiac edema is bimodal, with peaks appearing within 3 h and day 7 after MI (28). Therefore, LVA was induced in adult male mice according to previous protocols, with slight modifications. In this study, the heart in the LVA group was ligated at the LAD and the same level as the heart in the MI group; however, the suture was snipped on day 5 after the operation. At day 5 post-infarction, cardiac edema and inflammation increased. After snipping of the suture, left ventricular hemodynamic instability and the stress of the border zone increased; thus, left ventricular paradoxical movement appeared and LVA formation occurred.

The left ventricle in the LVA group was characterized by systolic dyskinesia and paradoxical bulging, whereas that in the MI group was only characterized by systolic dyskinesia. In this study, ADPN expression levels differed after coronary occlusion, leading to a significant difference in the lymphangiogenesis number and infiltration of LYVE1+ macrophages, thereby inducing different mouse models, such as MI and LVA. Mice administered ADPN had more cardiac lymphatic growth in the border zone than age-matched PBS-administered mice on day 28 after the operation, with improved heart function dependent on the administration time, which may be partly due to the expression or localization of Cx43 and macrophage status and abundance. The relationship between Cx43 and cardiac lymphatic growth is consistent with that reported in a previous study (29). Previous studies revealed that Cx37, Cx47, and Cx43 are the main connexin proteins that affect the developing lymphatic tissue (30). Mutations in these genes can lead to lymphatic disorders in mice and humans (31–33). In this study, Cx43 was expressed in the cardiac lymphatic vasculature during LVA formation following treatment with ADPN. Mouse models revealed that Cx43 expression affects not only lymphangiogenesis but also the maintenance of lymphatic function via LYVE1+ macrophages, which further strengthens our findings that ADPN affects Cx43+ LYVE1+ macrophages. Further studies are needed to fully elucidate how ADPN and connexins regulate these vessels, which may provide a therapeutic avenue to impede MI progression and LVA formation.

Cx43 dephosphorylation results in arrhythmia and cardiomyocyte apoptosis after cardiac ischemia/reperfusion (34). Macrophages facilitate electrical conduction in the heart via Cx43 expression (35). The decrease in oxygen in the cell lowers the pH and induces Cx43 degradation, resulting in MI-related complications, including arrhythmia (34, 36). To determine the effects of Cx43 on the injured myocardium following treatment with ADPN, verapamil and Gap19 were used. Verapamil was first used to treat hypertension and arrhythmias via the L-type calcium channels (37). However, verapamil indirectly affected Cx43 localization and stabilization (29, 38). During LVA formation, ADPN and verapamil synergistically enhanced LYVE1+ macrophages and lymphatic capillaries in the border zone (almost in the epicardial layer) via Cx43. Axin2 KO mice were used. Consistent activation of the Wnt/β-catenin signaling pathway was observed in adult mice (39), which also demonstrated the effects of Cx43 on LYVE1+ macrophages and lymphatic capillaries.

In this study, adipocyte-derived ADPN promoted macrophage M2a polarization and decreased inflammatory cytokine secretion via the ADPN/AdipoR2/HMGB1 axis; MMP and autophagy were lower in hypoxic HMGB1 knockdown macrophages than in non-knockdown macrophages, suggesting that HMGB1 is a key target of mitochondrial, autophagy, and inflammatory regulation. Hypoxia-reprogramed megamitochondrion contacted and engulfed lysosome to mediate mitochondrial self-digestion (40), which was consistent with the TEM results of the ADPN and ADPN+HMGB1 knockdown groups. According to Bushra et al., ADPN suppressed ROS production and apoptosis, and improved migration and barrier functions in hyperglycemic cells under 30 mM glucose conditions. Bioinformatics analysis revealed that the signaling pathways of integrin, HMGB1, STAT3, NFkB, and p38-AMPK were mainly involved in the actions of ADPN (41), which were consistent with our findings. ADPN promotes VEGF-C-dependent lymphangiogenesis through the AMPK/p38 signaling pathway (42, 43), which is consistent with the effects of ADPN on hypoxic macrophages.

ADPN decreased IL6, HMGB1, and C1qTNF9 secretion from macrophages, which may also be a senescence-associated secretory phenotype (SASP) (44). Macrophage senescence can partly explain the differences in immune responses after MI and the heterogeneity in the prognosis of patients with CAD. C1qTNF9 also regulates the fate of implanted mesenchymal stem cells to protect against MI injury (45, 46).

Macrophages are involved in all CAD stages. CD169+ macrophage or its “eat me” receptor Mertk deficiency impaired myocardial mitochondria elimination and cardiomyocyte-related hypoxic senescence in the early infarction period; therefore, ventricular functions decreased, and metabolic alterations appeared (47). MertK+ TREM2^high^ and MertK+ LYVE1+ macrophages negatively regulate inflammation and resolve lipid mediators related to the M2c anti-inflammatory effects (48). In addition, loss of CCR2^−^ macrophages led to the accumulation of the extracellular matrix component, hyaluronic acid (HA), which was needed to be cleared; this process required sensing by the HA receptor, LYVE-1 (49).

MI- or I/R-induced myocardial injury generates stress-induced senescent cells (SISCs), which play a critical role in the pathophysiology of adverse cardiac remodeling and heart failure by secreting proinflammatory molecules and matrix-degrading proteases (50). The removal of SISCs decreased inflammatory cytokines and normal cell death via macrophage efferocytosis. Abundant high-molecular-mass hyaluronic acid (HMM-HA) contributes to the longevity of the longest-lived rodents. HMM-HA also reduces inflammation during aging through several pathways, including a direct immunoregulatory effect on immune cells and protection from oxidative stress. LYVE1 on the cell membrane responds to HMM-HA in the matrix, alleviates senescence, and increases macrophage efferocytosis under stress (51). Therefore, LYVE1 may be a therapeutic target for the treatment of macrophage and cardiomyocyte senescence, and may improve the prognosis of patients with CAD. In our previous study, a CCR2 inhibitor was found to increase the ratio of CCR2^−^ macrophages, thereby strengthening the effects of adiponectin against myocardial injury after infarction (12). In this study, LNP-MertK was used to improve macrophage phagocytosis and heart function after MI by decreasing senescence. *In vivo*, transiently engineered CAR T-cell therapy delivered mRNA through LNP injection to reprogram T cells to recognize cardiac fibroblasts, thereby restoring heart function (52). We investigated whether this method could be used to treat macrophages after MI. Treatment with LNP-MertK increased the efficiency of endocytosis of the mitochondria and other unfit materials. Thus, LNP-MertK can be used to treat myocardial ischemia and/or reperfusion injury and increase the survival of cardiomyocytes to help outlive the early infarction period and decrease patient recurrent events. The effects of macrophage phagocytosis and senescence alleviation after LNP-MertK mRNA vaccine-like administration may be determined by evaluating migrasomes from macrophages (53, 54). Patients undergoing acute MI would survive longer if such treatment could be used in the early infarction period, which should be further investigated.

The crosstalk between adipocytes and macrophages is critical for disease progression. Adipocyte-derived lactate is a signaling metabolite that potentiates adipose macrophage inflammation by targeting PHD2 (55). Of note, intercellular mitochondrial transfer between adipocytes and macrophages is impaired in obesity (56). Macrophages limit the release of adipocyte mitochondria into the blood and cardiomyocytes; these effects are rescued during aging (57). In this study, IL6 and HMGB1 promoted ADPN expression in adipocytes via STAT3 and the co-transcription factor, YAP. The YAP/TEAD1 complex is a default repressor of the cardiac HMGB1/Toll-Like Receptor (TLR) axis (58) and may be a potential target for regulating the crosstalk between adipocytes and macrophages after MI. In this study, patients with CAD and obesity were divided into two groups based on lipid deposition and ADPN expression in the sub-epicardial adipocytes. The IL6/ADPN/HMGB1 axis was differentially expressed in human subepicardial tissue and blood samples, and was an independent factor in patients with CAD, which may partly explain the obesity paradox in patients that are overweight or obese, particularly among those who develop symptomatic CAD. However, BMI and other parameters of body composition are not consistent with the CAD risk factors for adverse short-term outcomes (59, 60). After treatment with high-thermogenic beige adipocytes (HBACs)-condition medium, the expression levels of antioxidant and anti-apoptotic genes were upregulated in H9c2 cardiomyocytes via NRF2 activation. Furthermore, HBAC-conditioned medium significantly attenuated ischemic rat heart tissue injury via NRF2 activation (61). Therefore, highly thermogenic beige adipocytes exert beneficial effects on cardiac injury by modulating NRF2 and are promising therapeutic agents for MI. The various modes of fat grafts after transplantation highlight the importance of macrophage-mediated ECM remodeling in graft preservation after fat grafting (62). This study may help preserve the retention rate of fat grafts.

This study had some limitations. First, more evidence and clearer explanations, especially regarding hemodynamic instability and border-zone stress, are needed for LVA formation. Second, intercellular mitochondrial transfer between adipocytes and macrophages, and mitochondrial and lipid droplet interactions, still need to be clearly elucidated in epicardial adipocyte ADPN-conditional knockout mice. Third, other leukocyte populations were not included. The effects of other target genes in RNA-seq analysis on Tregs or other leukocyte populations must be investigated using adipocyte-conditioned medium and conditional knockout mice. Finally, large sample sizes were not employed, and follow-up of the cohort was not conducted. The effects of the IL6/ADPN/HMGB1 axis must be examined in a large double-blind follow-up multicenter cohort.

## 5 Conclusion

Overall, ADPN impedes cardiac edema and promotes cardiac lymphangiogenesis by regulating Cx43. The IL6/STAT3/ADPN/HMGB1 axis contributes to the regulation of macrophage M2a polarization and sustained mitochondrial quality via crosstalk between adipocytes and macrophages in a mouse model. These findings were validated using human samples.

## Data availability statement

The datasets presented in this study can be found in online repositories. The names of the repository/repositories and accession number(s) can be found below: PRJNA1064646 (SRA).

## Ethics statement

This study was approved by the Ethics Committee of Tianjin Third Central Hospital. The studies were conducted in accordance with the local legislation and institutional requirements. The participants provided their written informed consent to participate in this study. This study was approved by the Ethics Committee of Nankai University (no. 2022-SYDWLL-000486). The study was conducted in accordance with the local legislation and institutional requirements.

## Author contributions

YZ: Writing – review & editing, Writing – original draft, Visualization, Methodology, Investigation, Data curation, Conceptualization. YW: Writing – review & editing, Investigation, Data curation. BQ: Writing – review & editing, Methodology, Investigation, Data curation. YL: Writing – review & editing, Resources, Methodology. ZZ: Writing – review & editing, Investigation. JM: Writing – review & editing, Validation, Resources. ML: Writing – review & editing, Validation, Resources. XL: Writing – review & editing, Visualization. YC: Writing – review & editing, Visualization. QZ: Writing – review & editing, Supervision. WG: Writing – review & editing, Supervision, Resources, Funding acquisition, Data curation. TL: Writing – review & editing, Supervision, Resources, Funding acquisition, Data curation.

## Supplementary material

Supplementary Figure 1 A simple, consistent, and low-cost LVA mice model construction. **(A)** Ligation site of heart organ in left coronary artery descending branch. **(B)** Intra-chamber cast and optical image of LVA (left) and MI (right), illustrating an outward bulge in the free wall of the left ventricle. **(C)** Optical LVA formation of mice heart: end-diastole (left) and end-systole (right) ligated at 4-4.5mm from its coronary origin. **(D)** Pattern picture of heart movement in LVA formation. **(E, F)** Coronal **(E)** and sagittal **(F)** position of computed tomography of LVA formation. SHAM and MI were 7d capture after model construction (n=6 per group). LVA formation was labeled with rectangle.

Supplementary Figure 2 LVA formation process. **(A)** Masson’s staining of representative images showed the LVA forming process in the total scenery (up, 25x) and the border regions (down, 100x) at 1d, 3d, 7d and 28d after operation. The SHAM and MI images at 7d after operation were as negative and positive control, respectively. Scale bar, 500μm and 100μm. **(B)** HE staining at border zone of representative images at the same d. Scale bar, 100μm. **(C)** Representative images of heart sections at border zone stained for cTNT (red) at the same day. Scale bar, 25μm. **(D–F)** Comparison of infarction area **(D)** and infarction size **(E)** in border zone compared to SHAM group. ** *P*<0.01, *** *P*<0.001, **** *P*<0.0001.

Supplementary Figure 3 Activation of pro-inflammatory and anti-inflammatory during LVA formation and progression. **(A)** The standard curve of the cytokine and chemokine measurements: IFN-ur GM-CSF, IL-1SF IL-6, IL-10, IL-22 and TNF22 **(B)** The concentrations of the cytokine and chemokine measurements: GM-CSF, IFNCS IL-1SF IL-6, IL-10, IL-22 and TNF2 among the three groups at 1d, 3d, 5d, 7d and 28d after operation (n=5 per group). LVA vs. MI: * *P*<0.05; ** *P*<0.01; *** *P*<0.001; **** *P*<0.0001.

Supplementary Figure 4 The survival rate among 3 groups. **(A)** The survival rate of LVA, MI and SHAM (n=25, 25 and 20, respectively). **(B)** Operation time of LVA, MI and SHAM (n=12 per group).

Supplementary Figure 5 ADPN regulated LYVE1+ macrophages and lymph-angiogenesis via Cx43 and Wnt canonical signaling. **(A)** The representative immunofluorescence images of Cx43 expression and Cx43 associated macrophages in the LVA mice treated with ADPN, verapamil and/or Gap19 from day 1 to 7. **(B)** The representative immunofluorescence images of Cx43 expression and Cx43 associated macrophages in WT or Axin2 KO LVA mice treated with ADPN and Gap19 from day 1 to 7, demonstrating that consistent activation of Wnt canonical signaling rescued the effects of downregulated Cx43 expression on Cx43 associated macrophages infiltration. **(C, D)** The representative immunofluorescence images **(C)** and quantity analysis **(D)** of LYVE1+ macrophages and lymph-angiogenesis in the LVA mice treated with ADPN, verapamil and/or Gap19 from day 1 to 7. **(E, F)** The representative immunofluorescence images **(E)** and quantity analysis **(F)** of LYVE1+ macrophages and lymph-angiogenesis in WT or Axin2 KO LVA mice treated with ADPN and Gap19 from day 1 to 7, demonstrating that consistent activation of Wnt canonical signaling rescued the effects of downregulated Cx43 expression. **(G)** The representative immunofluorescence images of Cx43 and ZO-1 expression and localization in the LVA mice treated with ADPN or control from day 1 to 7. **(H)** The mechanism diagram of MI and LVA formation. The ADPN expression level differed after coronary occlusion, leading to the significant difference of lymph-angiogenesis number and infiltrated LYVE1+ macrophages, thus inducing different mice model, such as MI and LVA formation. Scale bar, 500μm. * *P*<0.05; ** *P*<0.01; *** *P*<0.001; ns, not significant. Arrow, LYVE1+ macrophages. Scale bar, 500μm.

Supplementary Figure 6 HMGB1 knockdown led to mitochondrial quality control, autophagy reduction and M2b polarization in hypoxic macrophages. **(A)** qPCR and WB analysis demonstrated the HMGB1 knockdown efficiency in hypoxic macrophages. **(B)** ROS measurement in normal oxygen, hypoxia and HMGB1 knockdown Raw264.7. **(C)** Autophagy measurement in normal oxygen, hypoxia and HMGB1 knockdown Raw264.7 using MDC method. **(D)** The represented WB images of GPX4 and LC3 protein expression in normal oxygen, hypoxia and HMGB1 knockdown Raw264.7. **(E)** The related mRNA levels of IL1b, TNFa, IL6, Arg1, IL10 and Mrc1 in N, H and HMGB1 KD group. KD, knockdown; N, normal oxygen Raw264.7; H, hypoxia Raw264.7; HMGB1 KD, hypoxia HMGB1 knockdown Raw264.7. Scale bar, 100μm. *P<0.05; **P<0.01; ****P*<0.001.

Supplementary Figure 7 Supplying STAT3 overexpressed adipocytes decreased injury after LVA formation. **(A)** The HE representative images of heart sections in LVA and LVA+STAT3 overexpressed adipocytes groups. **(B)** The related wall thickness of border zone in LVA and LVA+STAT3 overexpressed adipocytes groups. **(C)** The border zone CKMB expression and LDH expression at 7d after operation in LVA and LVA+STAT3 overexpressed adipocytes groups using ELISA. Scale bar, 500μm. ***P<0.001.

Supplementary Figure 8 Aged macrophages hided in bone marrow. **(A)** The immunofluorescent images with b-gal and CD68 in bone marrow in young and aged MI mice. **(B)** Flow cytometry of F4/80 and CD206 staining to determine the macrophage M2 polarization in border zone of aged MI mice when treated LNP-MertK or LNP-MertK+ADPN at 7d after operation.

Supplementary Figure 9 Targeting MertK improved the heart functions after MI. **(A)** The representative echocardiogram image and quantity analysis, including LVEF and LVFS, in the SHAM, MI, MI+LNP and MI+LNP-MertK groups. **(B)** The representative images of Masson’s staining in the MI and MI+LNP-MertK groups. Scale bar, 500um. **(C)** HL-1 cardiomyocytes were pretreated with JC-1 and hypoxia 4h. Green labeled represented bad mitochondrial function. After that, transwell analysis was applied to cardiomyocytes (up) and macrophages, LNP treated macrophages or LNP-MertK treated macrophages (down). Nystatin was utilized to rescue the effects of LNP-MertK. Scale bar, 100um. **(D)** MitoTracker^®^ Red was used to pretreat hypoxic cardiomyocytes and transwell analysis and flow cytometry were used to analyze the protective effects of LNP-MertK on myocardial mitochondrial. **(E)** The macrophage polarization in four groups at 3d (up) and 7d (down) after operation. **(F)** The CKMB expression (up) and LDH expression (down) at 3d and 7d after MI using ELISA. **(G)** Flow cytometry of LYVE1 and MertK staining to determine the abundance of infiltrated phagocytic macrophages when treated with LNP-MertK or LNP-MertK+ADPN. **(H)** VEGFC expression was determined using ELISA at 3d and 7d after operation in the SHAM, MI, MI+LNP and MI+LNP-MertK groups. * P<0.05; **P<0.01; ***P<0.001; ns, not significant.

## References

[1] Naghavi M Abajobir AA Abbafati C Abbas KM Abd-Allah F Abera SF. Global, regional, and national age-sex specific mortality for 264 causes of death, 1980–2016: a systematic analysis for the Global Burden of Disease Study 2016. Lancet. (2017) 390:1151–210. doi: 10.1016/S0140-6736(17)32152-9 PMC5605883 28919116

[2] Chang J Liu X Sun Y. Mortality due to acutemyocardial infarction in China from 1987 to 2014: Secular trends and ageperiod-cohort effects. Int J Cardiol. (2017) 227:229–38. doi: 10.1016/j.ijcard.2016.11.130 27839815

[3] You J Wang X Wu J Gao L Wang X Du P. Predictors and prognosis of left ventricular thrombus in post-myocardial infarction patients with left ventricular dysfunction after percutaneous coronary intervention. J Thorac Dis. (2018) 10:4912–22. doi: 10.21037/jtd PMC6129917 30233865

[4] Sherrid MV Bernard S Tripathi N Patel Y Modi V Axel L. Apical aneurysms and mid-left ventricular obstruction in hypertrophic cardiomyopathy. JACC Cardiovasc Imaging. (2023) 16:591–605. doi: 10.1016/j.jcmg.2022.11.013 36681586

[5] Ruzza A Czer LSC Arabia F Vespignani R Esmailian F Cheng W. Left ventricular reconstruction for postinfarction left ventricular aneurysm: review of surgical techniques. Tex Heart Inst J. (2017) 44:326–35. doi: 10.14503/THIJ-16-6068 PMC5731585 29259502

[6] Michael H Mariaelvy B Donato S Remco TAM Jean-Yves S Irene N. Pericardial adipose tissue regulates granulopoiesis, fibrosis, and cardiac function after myocardial infarction. Circulation. (2018) 137:948–60. doi: 10.1161/CIRCULATIONAHA.117.028833 29167227

[7] Lu G Dina X Jing L Wayne BL Theodore AC Bernard L. Small extracellular microvesicles mediated pathological communications between dysfunctional adipocytes and cardiomyocytes as a novel mechanism exacerbating ischemia/reperfusion injury in diabetic mice. Circulation. (2020) 141:968–83. doi: 10.1161/CIRCULATIONAHA.119.042640 PMC7093230 31918577

[8] Elfeky M Kaede R Okamatsu-Ogura Y Kimura K. Adiponectin inhibits LPS-induced HMGB1 release through an AMP kinase and heme oxygenase-1-dependent pathway in RAW 264 macrophage cells. Mediators Inflamm.. (2016) 2016:5701959. doi: 10.1155/2016/5701959 27313399 PMC4904123

[9] Xia Y Zhang F Zhao S Li Y Chen X Gao E. Adiponectin determines farnesoid X receptor agonism-mediated cardioprotection against post-infarction remodelling and dysfunction. Cardiovasc Res. (2018) 114:1335–49. doi: 10.1093/cvr/cvy093 29668847

[10] Zhou B Honor LB He H Ma Q Oh JH Butterfield C. Adult mouse epicardium modulates myocardial injury by secreting paracrine factors. J Clin Invest. (2011) 121:1894–904. doi: 10.1172/JCI45529 PMC3083761 21505261

[11] Sun QN Wang YF Guo ZK. Reconstitution of myocardial lymphatic vessels after acute infarction of rat heart. Lymphology. (2012) 45:80–6. 23057153

[12] Zheng Y Gao W Qi B Zhang R Ning M Hu X. CCR2 inhibitor strengthens the adiponectin effects against myocardial injury after infarction. FASEB J. (2023) 37:e23039. doi: 10.1096/fj.202300281RR 37392374

[13] Takagawa J Zhang Y Wong ML Sievers RE Kapasi NK Wang Y. Myocardial infarct size measurement in the mouse chronic infarction model: comparison of area- and length-based approaches. J Appl Physiol (1985). (2007) 102:2104–11. doi: 10.1152/japplphysiol.00033.2007 PMC2675697 17347379

[14] Ashburner M Ball CA Blake JA Botstein D Butler H Cherry JM. Gene ontology: tool for the unification of biology. Gene Ontol Consortium Nat Genet. (2000) 25:25–9. doi: 10.1038/75556 PMC3037419 10802651

[15] Kanehisa M Goto S. KEGG: kyoto encyclopedia of genes and genomes. Nucleic Acids Res. (2000) 28:27–30. doi: 10.1093/nar/28.1.27 10592173 PMC102409

[16] Szklarczyk D Gable AL Lyon D Junge A Wyder S Huerta-Cepas J. STRING v11: protein-protein association networks with increased coverage, supporting functional discovery in genome-wide experimental datasets. Nucleic Acids Res. (2019) 47:D607–13. doi: 10.1093/nar/gky1131 PMC6323986 30476243

[17] Shannon P Markiel A Ozier O Baliga NS Wang JT Ramage D. Cytoscape: a software environment for integrated models of biomolecular interaction networks. Genome Res. (2003) 13:2498–504. doi: 10.1101/gr.1239303 PMC403769 14597658

[18] Tucker NR Chaffin M Fleming SJ Hall AW Parsons VA Bedi KC Jr. Transcriptional and cellular diversity of the human heart. Circulation. (2020) 142:466–82. doi: 10.1161/CIRCULATIONAHA.119.045401 PMC7666104 32403949

[19] Gonçalves C Ramalho MJ Silva R Silva V Marques-Oliveira R Silva AC. Lipid nanoparticles containing mixtures of antioxidants to improve skin care and cancer prevention. Pharmaceutics. (2021) 13:2042. doi: 10.3390/pharmaceutics13122042 34959324 PMC8706926

[20] Nicu C Jackson J Shahmalak A Pople J Ansell D Paus R. Adiponectin negatively regulates pigmentation, Wnt/β-catenin and HGF/c-Met signalling within human scalp hair follicles ex vivo. Arch Dermatol Res. (2023) 315:603–12. doi: 10.1007/s00403-021-02291-2 34854998

[21] Li Y Chen Y Yang T Chang K Deng N Zhao W. Targeting circulating high mobility group box-1 and histones by extracorporeal blood purification as an immunomodulation strategy against critical illnesses. Crit Care. (2023) 27:77. doi: 10.1186/s13054-023-04382-0 36855150 PMC9972334

[22] Parikh NI Gona P Larson MG Fox CS Benjamin EJ Murabito JM. Long-term trends in myocardial infarction incidence and case fatality in the National Heart, Lung, and Blood Institute's Framingham Heart study. Circulation. (2009) 119:1203–10. doi: 10.1161/CIRCULATIONAHA.108.825364 PMC2725400 19237656

[23] Yan X Zhang H Fan Q Hu J Tao R Chen Q. Dectin-2 deficiency modulates th1 differentiation and improves wound healing after myocardial infarction. Circ Res. (2017) 120:1116–29. doi: 10.1161/CIRCRESAHA.116.310260 28193608

[24] Jackson BM Gorman JH III Moainie SL Guy TS Narula N Narula J. Extension of borderzone myocardium in postinfarction dilated cardiomyopathy. J Am Col Cardiol. (2002) 40:1160–7. doi: 10.1016/S0735-1097(02)02121-6 12354444

[25] Ratcliffe MB. Non-ischemic infarct extension: A new type of infarct enlargement and a potential therapeutic target. J Am Col Cardiol. (2002) 40:1168–71. doi: 10.1016/S0735-1097(02)02113-7

[26] Liu S Yin T Wei X Yi W Qu Y Liu Y. Downregulation of adiponectin induced by tumor necrosis factor α is involved in the aggravation of posttraumatic myocardial ischemia/reperfusion injury. Crit Care Med. (2011) 39:1935–43. doi: 10.1097/CCM.0b013e31821b85db 21499085

[27] Gao C Liu Y Yu Q Yang Q Li B Sun L. TNF-α antagonism ameliorates myocardial ischemia-reperfusion injury in mice by upregulating adiponectin. Am J Physiol Heart Circ Physiol. (2015) 308:H1583–1591. doi: 10.1152/ajpheart.00346.2014 25888509

[28] Fernandez-Jimenez R Sanchez-Gonzalez J Aguero J Garcia-Prieto J Lopez-Martin GJ Garcia-Ruiz JM. Myocardial edema after ischemia/reperfusion is not stable and follows a bimodal pattern: imaging and histological tissue characterization. J Am Coll Cardiol. (2015) 65:315–23. doi: 10.1016/j.jacc.2014.11.004 25460833

[29] Trincot CE Xu W Zhang H Kulikauskas MR Caranasos TG Jensen BC. Adrenomedullin induces cardiac lymphangiogenesis after myocardial infarction and regulates cardiac edema *via* connexin 43. Circ Res. (2019) 124:101–13. doi: 10.1161/CIRCRESAHA.118.313835 PMC6318063 30582443

[30] Munger SJ Geng X Srinivasan RS Witte MH Paul DL Simon AM. Segregated Foxc2, NFATc1 and Connexin expression at normal developing venous valves, and Connexin-specific differences in the valve phenotypes of Cx37, Cx43, and Cx47 knockout mice. Dev Biol. (2016) 412:173–90. doi: 10.1016/j.ydbio.2016.02.033 PMC4826804 26953188

[31] Kanady JD Dellinger MT Munger SJ Witte MH Simon AM. Connexin37 and Connexin43 deficiencies in mice disrupt lymphatic valve development and result in lymphatic disorders including lymphedema and chylothorax. Dev Biol. (2011) 354:253–66. doi: 10.1016/j.ydbio.2011.04.004 PMC3134316 21515254

[32] Munger SJ Davis MJ Simon AM. Defective lymphatic valve development and chylothorax in mice with a lymphatic-specific deletion of Connexin43. Dev Biol. (2017) 421:204–18. doi: 10.1016/j.ydbio.2016.11.017 PMC5217530 27899284

[33] Ferrell RE Baty CJ Kimak MA Karlsson JM Lawrence EC Franke-Snyder M. GJC2 missense mutations cause human lymphedema. Am J Hum Genet. (2010) 86:943–8. doi: 10.1016/j.ajhg.2010.04.010 PMC3032064 20537300

[34] Jingyi X Xinxin Y Yutong Y Min C Lulin W Zhongshan G. Connexin 43 dephosphorylation contributes to arrhythmias and cardiomyocyte apoptosis in ischemia/reperfusion hearts. Basic Res Cardiol. (2019) 114(5):40. doi: 10.1007/s00395-019-0748-8 31463533

[35] Maarten H Sebastian C Ling X Aguirre AD King KR Hanley A. Macrophages facilitate electrical conduction in the heart. Cell. (2017) 169:510–522.e20. doi: 10.1016/j.cell.2017.03.050 28431249 PMC5474950

[36] Kieken F Mutsaers N Dolmatova E Virgil K Wit AL Kellezi A. Structural and molecular mechanisms of gap junction remodeling in epicardial border zone myocytes following myocardial infarction. Circ Res. (2009) 104:1103–12. doi: 10.1161/CIRCRESAHA.108.190454 PMC2892879 19342602

[37] Curtis MJ Walker MJ. The mechanism of action of the optical enantiomers of verapamil against ischaemia-induced arrhythmias in the conscious rat. Br J Pharmacol. (1986) 89:137–47. doi: 10.1111/j.1476-5381.1986.tb11129.x PMC1917029 3801768

[38] Zhou P Zhang SM Wang QL Wu Q Chen M Pei JM. Anti-arrhythmic effect of verapamil is accompanied by preservation of cx43 protein in rat heart. PloS One. (2013) 8:e7156710. doi: 10.1371/journal.pone.0071567 PMC3741134 23951191

[39] Yan Y Tang D Chen M Huang J Xie R Jonason JH. Axin2 controls bone remodeling through the beta-catenin-BMP signaling pathway in adult mice. J Cell Sci. (2009) 122:3566–78. doi: 10.1242/jcs.051904 PMC2746134 19737815

[40] Hao T Yu J Wu Z Jiang J Gong L Wang B. Hypoxia-reprogramed megamitochondrion contacts and engulfs lysosome to mediate mitochondrial self-digestion. Nat Commun. (2023) 14:4105. doi: 10.1038/s41467-023-39811-9 37433770 PMC10336010

[41] Bushra S Al-Sadeq DW Bari R Sahara A Fadel A Rizk N. Adiponectin ameliorates hyperglycemia-induced retinal endothelial dysfunction, highlighting pathways, regulators, and networks. J Inflamm Res. (2022) 15:3135–66. doi: 10.2147/JIR.S358594 PMC9156523 35662872

[42] Huang CY Chang AC Chen HT Wang SW Lo YS Tang CH. Adiponectin promotes VEGF-C-dependent lymphangiogenesis by inhibiting miR-27b through a CaMKII/AMPK/p38 signaling pathway in human chondrosarcoma cells. Clin Sci (Lond). (2016) 130:1523–33. doi: 10.1042/CS20160117 27252405

[43] Wang Y Liang B Lau WB Du Y Guo R Yan Z. Restoring diabetes-induced autophagic flux arrest in ischemic/reperfused heart by ADIPOR (adiponectin receptor) activation involves both AMPK-dependent and AMPK-independent signaling. Autophagy. (2017) 13:1855–69. doi: 10.1080/15548627.2017.1358848 PMC5788496 28825851

[44] Di Micco R Krizhanovsky V Baker D d'Adda di Fagagna F. Cellular senescence in ageing: from mechanisms to therapeutic opportunities. Nat Rev Mol Cell Biol. (2021) 22:75–95. doi: 10.1038/s41580-020-00314-w 33328614 PMC8344376

[45] Yan W Guo Y Tao L Lau WB Gan L Yan Z. C1q/tumor necrosis factor-related protein-9 regulates the fate of implanted mesenchymal stem cells and mobilizes their protective effects against ischemic heart injury *via* multiple novel signaling pathways. Circulation. (2017) 136:2162–77. doi: 10.1161/CIRCULATIONAHA.117.029557 PMC5705403 28978553

[46] Liu D Gu G Gan L Yan W Zhang Z Yao P. Identification of a CTRP9 C-Terminal polypeptide capable of enhancing bone-derived mesenchymal stem cell cardioprotection through promoting angiogenic exosome production. Redox Biol. (2021) 41:101929. doi: 10.1016/j.redox.2021.101929 33714738 PMC7966869

[47] Nicolás-Ávila JA Lechuga-Vieco AV Esteban-Martínez L Sánchez-Díaz M Díaz-García E Santiago DJ. A network of macrophages supports mitochondrial homeostasis in the heart. Cell. (2020) 183:94–109.e23. doi: 10.1016/j.cell.2020.08.031 32937105

[48] Alivernini S MacDonald L Elmesmari A Finlay S Tolusso B Gigante MR. Distinct synovial tissue macrophage subsets regulate inflammation and remission in rheumatoid arthritis. Nat Med. (2020) 26:1295–306. doi: 10.1038/s41591-020-0939-8 32601335

[49] Voisin B Nadella V Doebel T Goel S Sakamoto K Ayush O. Macrophage-mediated extracellular matrix remodeling controls host Staphylococcus aureus susceptibility in the skin. Immunity. (2023) 56:1561–1577.e9. doi: 10.1016/j.immuni.2023.06.006 37402364 PMC10467568

[50] Lee JR Park BW Park JH Lim S Kwon SP Hwang JW. Local delivery of a senolytic drug in ischemia and reperfusion-injured heart attenuates cardiac remodeling and restores impaired cardiac function. Acta Biomater. (2021) 135:520–33. doi: 10.1016/j.actbio.2021.08.028 34454081

[51] Zhang Z Tian X Lu JY Boit K Ablaeva J Zakusilo FT. Increased hyaluronan by naked mole-rat Has2 improves healthspan in mice. Nature. (2023) 621:196–205. doi: 10.1038/s41586-023-06463-0 37612507 PMC10666664

[52] Rurik JG Tombácz I Yadegari A Méndez Fernández PO Shewale SV Li L. CAR T cells produced in vivo to treat cardiac injury. Science. (2022) 375:91–6. doi: 10.1126/science.abm0594 PMC9983611 34990237

[53] Jiao H Jiang D Hu X Du W Ji L Yang Y. Mitocytosis, a migrasome-mediated mitochondrial quality-control process. Cell. (2021) 184:2896–2910.e13. doi: 10.1016/j.cell.2021.04.027 34048705

[54] Zheng Y Lang Y Qi B Li T. TSPAN4 and migrasomes in atherosclerosis regression correlated to myocardial infarction and pan-cancer progression. Cell Adh Migr. (2023) 17:14–9. doi: 10.1080/19336918.2022.2155337 PMC9754108 36513632

[55] Feng T Zhao X Gu P Yang W Wang C Guo Q. Adipocyte-derived lactate is a signalling metabolite that potentiates adipose macrophage inflammation *via* targeting PHD2. Nat Commun. (2022) 13:5208. doi: 10.1038/s41467-022-32871-3 36064857 PMC9445001

[56] Brestoff JR Wilen CB Moley JR Li Y Zou W Malvin NP. Intercellular mitochondria transfer to macrophages regulates white adipose tissue homeostasis and is impaired in obesity. Cell Metab. (2021) 33:270–282.e8. doi: 10.1016/j.cmet.2020.11.008 33278339 PMC7858234

[57] Borcherding N Jia W Giwa R Field RL Moley JR Kopecky BJ. Dietary lipids inhibit mitochondria transfer to macrophages to divert adipocyte-derived mitochondria into the blood. Cell Metab. (2022) 34:1499–1513.e8. doi: 10.1016/j.cmet.2022.08.010 36070756 PMC9547954

[58] Gao Y Sun Y Ercan-Sencicek AG King JS Akerberg BN Ma Q. YAP/TEAD1 complex is a default repressor of cardiac toll-like receptor genes. Int J Mol Sci. (2021) 22:6649. doi: 10.3390/ijms22136649 34206257 PMC8268263

[59] Julia R. How the U.S. Low-Fat Diet Recommendations of 1977 Contributed to the Declining Health of Americans. Honors Scholar Theses. (2016). p. 490.

[60] Powell-Wiley TM Poirier P Burke LE Després JP Gordon-Larsen P Lavie CJ. Obesity and cardiovascular disease: A scientific statement from the american heart association. Circulation. (2021) 143:e984–e1010. doi: 10.1161/CIR.0000000000000973 33882682 PMC8493650

[61] Moon H Choi JW Song BW Kim IK Lim S Lee S. Brite adipocyte FGF21 attenuates cardiac ischemia/reperfusion injury in rat hearts by modulating NRF2. Cells. (2022) 11:567. doi: 10.3390/cells11030567 35159376 PMC8833946

[62] Zhang X Jin X Li Y Xu M Yao Y Liu K. Macrophage-mediated extracellular matrix remodeling after fat grafting in nude mice. FASEB J. (2022) 36:e22550. doi: 10.1096/fj.202200037R 36098482

