# Supplementary material for: IL6/adiponectin/HMGB1 feedback loop mediates adipocyte and macrophage crosstalk and M2 polarization after myocardial infarction
Source: Front Immunol. 2024 Mar 27;15:1368516. doi: 10.3389/fimmu.2024.1368516 (PMC11004445; doi:10.3389/fimmu.2024.1368516)
Supplement: Supplementary file 1 [file Table_1.docx]

Supplementary Table 1. Specific primers used for quantitative real-time PCR.

| Genes | Forward (5’-3’) | Reverse (5’-3’) |
| --- | --- | --- |
| HMGB1 | GGCGAGCATCCTGGCTTATC | GGCTGCTTGTCATCTGCTG |
| ADPN | TGTTCCTCTTAATCCTGCCCA | CCAACCTGCACAAGTTCCCTT |
| AdipoR1 | AGACAACGACTACCTGCTACA | GTGGATGCGGAAGATGCTCT |
| AdipoR2 | GGAGTGTTCGTGGGCTTAGG | GCAGCTCCGGTGATATAGAGG |
| IL6 | TAGTCCTTCCTACCCCAATTTCC | TTGGTCCTTAGCCACTCCTTC |
| Arg-1 | CTCCAAGCCAAAGTCCTTAGAG | AGGAGCTGTCATTAGGGACATC |
| IL-10 | CCCATTCCTCGTCACGATCTC | TCAGACTGGTTTGGGATAGGTTT |
| Mrc-1 | CTCTGTTCAGCTATTGGACGC | CGGAATTTCTGGGATTCAGCTTC |
| IL-1B | GCAACTGTTCCTGAACTCAACT | ATCTTTTGGGGTCCGTCAACT |
| TNFa | CCCTCACACTCAGATCATCTTCT | GCTACGACGTGGGCTACAG |
| B-actin | GGCTGTATTCCCCTCCATCG | CCAGTTGGTAACAATGCCATGT |

Supplementary Table 2. Specific primers used for ChIP-PCR.

| Genes | Forward (5’-3’) | Reverse (5’-3’) |
| --- | --- | --- |
| ADPN promoter 1 | TCACAATGCTCCCGGGTGTCT | CAGATACCTGTGGTTGTCTCC |
| ADPN promoter 2 | TCTAGCTTCGTGCCTTGGGTC | AGCAACTCAAAGCTGTATCAC |
| ADPN promoter 3 | GACTCGACTACCCCTTGACTC | TACTAGTGGATAGGACCAGCA |
| ADPN promoter 4 | AGCCCTTGGAAATGAGCTTGT | CACCACTCACTAAACTGCTCC |
| ADPN promoter 5 | TCTTATTGGAGCAGCTGCTGG | TAAAATCTGTGGTCCCTACAT |
| ADPN promoter 6 | TAAGTGTTCTGTGACACTGGG | AAAAAATAGATGGACTGCAGG |
| ADPN promoter 7 | TTCTCAGTATGGGATCCGGTC | AATTCAGCATGTTTCTGAGTG |
| ADPN promoter 8 | TGCTCTTAACCACTGAGCCAT | AAATGAGGAGAAAGTGGCCAG |
| B-actin | GGCTGTATTCCCCTCCATCG | CCAGTTGGTAACAATGCCATGT |

Supplementary Table 3. The significant GO and KEGG pathways enriched by DEGs between MI 7d and LVA 7d border zone.

| Ontology | ID | Description | GeneRatio | BgRatio | pvalue | p.adjust | zscore |
| --- | --- | --- | --- | --- | --- | --- | --- |
| BP | GO:1990266 | neutrophil migration | 12/104 | 130/28814 | 5.15e-14 | 8.74e-11 | -3.4641 |
| BP | GO:0030593 | neutrophil chemotaxis | 11/104 | 103/28814 | 1.19e-13 | 1.01e-10 | -3.3166 |
| BP | GO:0050900 | leukocyte migration | 16/104 | 380/28814 | 5.65e-13 | 2.48e-10 | -4 |
| BP | GO:0097530 | granulocyte migration | 12/104 | 159/28814 | 5.84e-13 | 2.48e-10 | -3.4641 |
| BP | GO:0071621 | granulocyte chemotaxis | 11/104 | 128/28814 | 1.35e-12 | 4.58e-10 | -3.3166 |
| CC | GO:0034364 | high-density lipoprotein particle | 3/102 | 38/28739 | 0.0003 | 0.0245 | -1.7321 |
| CC | GO:0034358 | plasma lipoprotein particle | 3/102 | 47/28739 | 0.0006 | 0.0245 | -1.7321 |
| CC | GO:1990777 | lipoprotein particle | 3/102 | 47/28739 | 0.0006 | 0.0245 | -1.7321 |
| CC | GO:0032994 | protein-lipid complex | 3/102 | 50/28739 | 0.0008 | 0.0245 | -1.7321 |
| CC | GO:0016459 | myosin complex | 3/102 | 54/28739 | 0.0009 | 0.0245 | -1.7321 |
| MF | GO:0005125 | cytokine activity | 12/102 | 228/28275 | 4.03e-11 | 1.06e-08 | -2.8868 |
| MF | GO:0045236 | CXCR chemokine receptor binding | 4/102 | 15/28275 | 2.11e-07 | 1.99e-05 | -2 |
| MF | GO:0005126 | cytokine receptor binding | 10/102 | 316/28275 | 2.27e-07 | 1.99e-05 | -3.1623 |
| MF | GO:0008009 | chemokine activity | 5/102 | 41/28275 | 3.74e-07 | 2.46e-05 | -2.2361 |
| MF | GO:0042379 | chemokine receptor binding | 5/102 | 77/28275 | 8.9e-06 | 0.0005 | -2.2361 |
| KEGG | mmu04060 | Cytokine-cytokine receptor interaction | 13/47 | 292/9000 | 1.77e-09 | 2.09e-07 | -3.6056 |
| KEGG | mmu04657 | IL-17 signaling pathway | 8/47 | 93/9000 | 2.17e-08 | 1.28e-06 | -2.8284 |
| KEGG | mmu05323 | Rheumatoid arthritis | 7/47 | 87/9000 | 2.84e-07 | 1.12e-05 | -2.6458 |
| KEGG | mmu04061 | Viral protein interaction with cytokine and cytokine receptor | 6/47 | 95/9000 | 8.94e-06 | 0.0003 | -2.4495 |
| KEGG | mmu05146 | Amoebiasis | 6/47 | 107/9000 | 1.77e-05 | 0.0004 | -2.4495 |

DEGs, Different Expressed Genes; GO, Gene ONTOLOGY; BP, Biological Process; CC, cellular component; MF, Molecular Function; KEGG, Kyoto Encyclopedia of Genes and Genomes.

Supplementary Table 4. Effect of Adiponectin treatment during different periods on cardiac healing after LVA in mice.

|  | LV Vol; d  (μL) | LV Vol; s  (μL) | LVEF (%) | LVFS (%) | LV Mass (AW, mg) |
| --- | --- | --- | --- | --- | --- |
| **Adiponectin (1-14)** |  |  |  |  |  |
| Adiponectin (day3) | 65.7±3.3* | 40.5±2.5* | 38.3±2.1* | 19.1±1.0* | 105.2±4.2* |
| PBS (day3) | 76.2±3.6 | 53.8±3.2 | 29.7±2.5 | 12.7±1.4 | 115.4±5.6 |
| Adiponectin (day7) | 65.6±4.2* | 42.9±3.6* | 34.7±2.5* | 16.2±1.3* | 95.3±7.4* |
| PBS (day7) | 79.7±6.8 | 60.5±4.5 | 24.3±4.0 | 10.7±2.1 | 110.9±11.2 |
| Adiponectin (day28) | 59.5±5.4* | 47.0±4.3 | 21.0±2.6* | 9.5±1.5* | 129.6±6.7* |
| PBS (day28) | 71.0±5.2 | 49.3±3.1 | 30.7±2.9 | 14.3±1.6 | 115.7±6.5 |
|  |  |  |  |  |  |
| **Adiponectin (1-3)** |  |  |  |  |  |
| Adiponectin (day3) | 65.3±3.1* | 40.7±2.6* | 38.7±2.5* | 19.3±1.4* | 106.3±4.6* |
| PBS (day3) | 72.0±2.9 | 50.9±2.4 | 29.3±2.5 | 12.4±1.4 | 127.5±5.2 |
| Adiponectin (day7) | 65.7±3.7* | 45.9±2.9* | 30.3±2.1* | 14.2±1.0* | 107.5±5.9* |
| PBS (day7) | 73.3±4.1 | 56.8±3.1 | 22.7±2.1 | 10.2±1.1 | 127.0±4.1 |
| Adiponectin (day28) | 62.7±2.9* | 41.2±2.5* | 34.3±2.5* | 16.0±1.4* | 98.4±3.6* |
| PBS (day28) | 68.4±3.8 | 49.5±2.7 | 27.7±1.3 | 11.5±0.7 | 112.7±8.6 |
|  |  |  |  |  |  |
| **Adiponectin (1-7)** |  |  |  |  |  |
| Adiponectin (day3) | 60.7±2.9* | 37.5±3.2* | 38.3±1.5* | 19.0±0.8* | 108.4±4.7* |
| PBS (day3) | 73.2±4.2 | 50.8±4.1 | 30.7±1.5 | 14.4±0.7 | 122.6±4.3 |
| Adiponectin (day7) | 70.3±3.3* | 45.7±2.6* | 34.8±2.6* | 16.4±1.5* | 111.3±4.2* |
| PBS (day7) | 79.8±4.7 | 59.6±2.8 | 25.3±2.5 | 11.2±1.4 | 128.7±8.3 |
| Adiponectin (day28) | 66.7±4.0* | 39.8±3.4* | 40.7±1.4* | 20.3±0.6* | 115.2±2.6* |
| PBS (day28) | 77.2±3.5 | 54.4±2.8 | 29.7±2.6 | 12.6±1.6 | 126.6±5.7 |
|  |  |  |  |  |  |
| **Adiponectin (3-14)** |  |  |  |  |  |
| Adiponectin (day3) | 69.1±2.4 | 45.5±2.4 | 34.2±1.6 | 15.8±0.7 | 110.8±3.1 |
| PBS (day3) | 68.7±2.1 | 45.8±2.3 | 33.4±1.4 | 15.7±0.6 | 106.7±5.2 |
| Adiponectin (day7) | 84.5±3.2* | 59.4±2.9 | 29.7±1.5* | 12.5±0.7* | 112.8±9.9* |
| PBS (day7) | 76.3±3.6 | 59.2±2.2 | 22.2±1.8 | 9.9±0.9 | 129.0±4.6 |
| Adiponectin (day28) | 79.9±2.6* | 62.9±3.2* | 21.3±1.6* | 9.6±0.7* | 129.1±6.9* |
| PBS (day28) | 71.7±1.9 | 46.7±3.5 | 35.0±1.9 | 16.6±0.9 | 112.4±7.4 |
|  |  |  |  |  |  |
| **Adiponectin (7-14)** |  |  |  |  |  |
| Adiponectin (day3) | 75.6±4.3 | 50.5±4.3* | 32.7±2.1* | 15.4±1.2* | 114.3±4.5* |
| PBS (day3) | 79.4±4.6 | 56.9±3.8 | 28.3±1.5 | 12.1±0.7 | 127.4±6.9 |
| Adiponectin (day7) | 76.3±3.7 | 57.5±3.5 | 24.7±1.6 | 10.0±0.8 | 128.8±8.5 |
| PBS (day7) | 77.8±4.1 | 60.7±4.0 | 22.3±2.1 | 9.8±1.1 | 127.9±4.4 |
| Adiponectin (day28) | 86.5±2.6* | 70.3±3.0* | 18.7±1.6* | 8.6±0.8* | 138.9±5.9* |
| PBS (day28) | 78.9±3.1 | 54.7±3.3 | 30.7±1.5 | 14.7±0.6 | 123.1±3.7 |

LV Vol; d: left ventricular diastolic volume; LVID, s: left ventricular systolic volume; LVEF: left ventricular ejection fraction; FS: left ventricular fractional shortening; LV Mass: left ventricular mass.

* P< 0.05, vs. PBS group.

Supplementary Table 5. The characteristics of young, middle, and aged CABG patients.

| Characteristics | young | middle | aged | P value |
| --- | --- | --- | --- | --- |
| n | 38 | 62 | 34 |  |
| gender, n (%) |  |  |  | 0.146 |
| male | 26 (19.4%) | 56 (41.8%) | 28 (20.9%) |  |
| female | 12 (9%) | 6 (4.5%) | 6 (4.5%) |  |
| smoke, n (%) |  |  |  | 0.926 |
| no | 13 (10.4%) | 22 (16.4%) | 14 (10.4%) |  |
| yes | 24 (17.9%) | 40 (29.9%) | 20 (14.9%) |  |
| Diabetes, year | 1 (0, 7.5) | 1 (0, 8) | 1 (0, 2) | 0.712 |
| encephalopathy, n (%) |  |  |  | 0.503 |
| no | 28 (20.9%) | 36 (26.9%) | 20 (14.9%) |  |
| yes | 10 (7.5%) | 26 (19.4%) | 14 (10.4%) |  |
| CAD history, n (%) |  |  |  | 0.476 |
| no | 34 (25.4%) | 44 (32.8%) | 24 (17.9%) |  |
| yes | 4 (3%) | 18 (13.4%) | 10 (7.5%) |  |
| PCI or CABG, n (%) |  |  |  | 0.180 |
| no | 36 (26.9%) | 46 (34.3%) | 26 (19.4%) |  |
| yes | 2 (1.5%) | 16 (11.9%) | 8 (6%) |  |
| Systolic pressure, mmHg | 117.63 ± 21.292 | 131.68 ± 18.258 | 138.18 ± 22.023 | 0.009 |
| diatolic pressure, mmHg | 71.263 ± 11.155 | 74.774 ± 7.3562 | 78 ± 7.7298 | 0.074 |
| single or not, n (%) |  |  |  | 0.811 |
| multivessel | 32 (23.9%) | 56 (41.8%) | 30 (22.4%) |  |
| single vessel | 6 (4.5%) | 6 (4.5%) | 4 (3%) |  |
| culprit vessel, n (%) |  |  |  | 0.088 |
| LAD | 26 (19.4%) | 24 (17.9%) | 14 (10.4%) |  |
| RCA | 10 (7.5%) | 34 (25.4%) | 12 (9%) |  |
| LCX | 2 (1.5%) | 4 (3%) | 2 (1.5%) |  |
| LCA | 0 (0%) | 0 (0%) | 4 (3%) |  |
| D1 | 0 (0%) | 0 (0%) | 2 (1.5%) |  |
| treatment, n (%) |  |  |  | 0.261 |
| PTCA | 20 (14.9%) | 26 (19.4%) | 12 (9%) |  |
| PCI | 12 (9%) | 28 (20.9%) | 22 (16.4%) |  |
| CABG | 6 (4.5%) | 8 (6%) | 0 (0%) |  |
| TG, mmol/L | 1.26 (1.03, 1.645) | 1.08 (0.93, 1.74) | 2.15 (1.4225, 3.3425) | 0.006 |
| LDL, mmol/L | 2.6805 ± 0.91645 | 2.8193 ± 0.85517 | 3.1379 ± 0.85369 | 0.330 |
| HDL, mmol/L | 1 (0.88, 1.19) | 0.98 (0.89, 1.215) | 1.03 (0.8325, 1.1575) | 0.946 |
| TC, mmol/L | 4.4647 ± 1.2171 | 4.2585 ± 1.2485 | 4.9507 ± 1.6591 | 0.301 |
| LDH, mmol/L | 378 (294.5, 481) | 376 (292, 538) | 500.5 (332, 655.5) | 0.463 |
| HBDH, mmol/L | 366 (271, 438.5) | 357 (271, 414) | 462 (300.75, 630.5) | 0.613 |
| CK, ng/ml | 756 (419, 924) | 685 (203, 1168.5) | 812 (659, 2248) | 0.449 |
| CK-MB, ng/ml | 71 (35, 83.5) | 72 (22, 118) | 98 (61, 180) | 0.466 |
| cTNI, ng/ml | 9.33 (6.205, 26.305) | 11.4 (4.85, 19.77) | 13.76 (9.44, 33.6) | 0.482 |
| BNP, pg/ml | 133 (45.2, 226.95) | 102 (48.45, 157.55) | 68.3 (38.3, 115) | 0.270 |
| Uric Acid, mmol/L | 320 (268.5, 385.5) | 336 (260, 393.5) | 310 (259, 362) | 0.754 |
| serum creatinine, µmol/L | 71 (66, 84) | 74 (64.5, 86.5) | 69 (57, 80) | 0.206 |
| Fasting venous glucose, mmol/L | 7.6 (5.33, 9.925) | 7.29 (6.23, 8.595) | 7.19 (5.97, 13.56) | 0.787 |
| glycosylated hemoglobin, mmol/L | 7.1 (6.05, 8.75) | 6.7 (5.85, 9.8) | 7.4 (6.3, 9.9) | 0.682 |
| albumin, g/L | 37.158 ± 4.7948 | 39.897 ± 3.5547 | 40.6 ± 3.9397 | 0.025 |
| globulin, mg/L | 24.532 ± 4.6227 | 23.029 ± 4.183 | 24.865 ± 3.8977 | 0.279 |
| TBIL, µmol/L | 10.8 (8.7, 13.5) | 14.1 (10, 16.6) | 13.3 (10.8, 18.9) | 0.215 |
| direct bilirubin, µmol/L | 1.7421 ± 0.81875 | 1.6935 ± 0.72293 | 2.0294 ± 0.9184 | 0.369 |
| indirect bilirubin, µmol/L | 9.1 (8.05, 12) | 11.5 (8.4, 14.4) | 9.9 (8.5, 15.3) | 0.323 |
| ALP, U/L | 73 (54.5, 89) | 72 (61.5, 84.5) | 83 (71, 103) | 0.070 |
| ALT, U/L | 28 (16, 51) | 27 (21.5, 52.5) | 38 (20, 60) | 0.643 |
| AST, U/L | 117 (56.5, 197) | 126 (42.5, 184.5) | 101 (24, 235) | 0.870 |
| ADPN, pg/mL | 172.37 (164.14, 177.56) | 200.15 (190.75, 207.25) | 234.47 (215.61, 302.75) | < 0.001 |
| IL6, pg/mL | 140.83 (134.34, 162.44) | 116.44 (108.73, 120.26) | 86.55 (71.94, 97.05) | < 0.001 |
| HMGB1, pg/mL | 154.1 (140.99, 186.7) | 109.16 (101.5, 120.84) | 83.31 (79.61, 87.57) | < 0.001 |

Supplementary Table 6. The logistic analysis of young, middle, and aged CABG patients.

| Characteristics | Total(N) | Univariate analysis | |  | Multivariate analysis | |
| --- | --- | --- | --- | --- | --- | --- |
|  |  | Odds Ratio (95% CI) | P value |  | Odds Ratio (95% CI) | P value |
| Systolic pressure | 96 | 1.044 (1.011 - 1.077) | **0.008** |  | 1.034 (0.997 - 1.072) | 0.071 |
| TG | 82 | 1.380 (1.010 - 1.886) | **0.043** |  | 1.402 (1.033 - 1.904) | **0.030** |
| albumin | 89 | 1.200 (1.038 - 1.386) | **0.013** |  | 1.188 (1.005 - 1.405) | **0.043** |
| ALP | 89 | 1.027 (1.003 - 1.053) | **0.030** |  | 1.022 (0.993 - 1.051) | 0.137 |
| ADPN | 96 | 1.136 (1.223 - 1.398) | **0.010** |  | 1.005 (0.982 - 1.029) | 0.662 |
| IL6 | 96 | 0.983 (0.967 - 1.000) | **0.050** |  | 0.980 (0.926 - 1.038) | 0.492 |
| HMGB1 | 96 | 0.988 (0.975 - 1.001) | 0.070 |  | 0.994 (0.949 - 1.040) | 0.790 |
| ADPN/(IL6*HMGB1) | 96 | 2.034 (1.997 - 2.072) | **0.002** |  | 1.044 (1.011 - 1.077) | **0.008** |
