# Supplementary figures and images for: IL6/adiponectin/HMGB1 feedback loop mediates adipocyte and macrophage crosstalk and M2 polarization after myocardial infarction
Source: Front Immunol. 2024 Mar 27;15:1368516. doi: 10.3389/fimmu.2024.1368516 (PMC11004445; doi:10.3389/fimmu.2024.1368516)

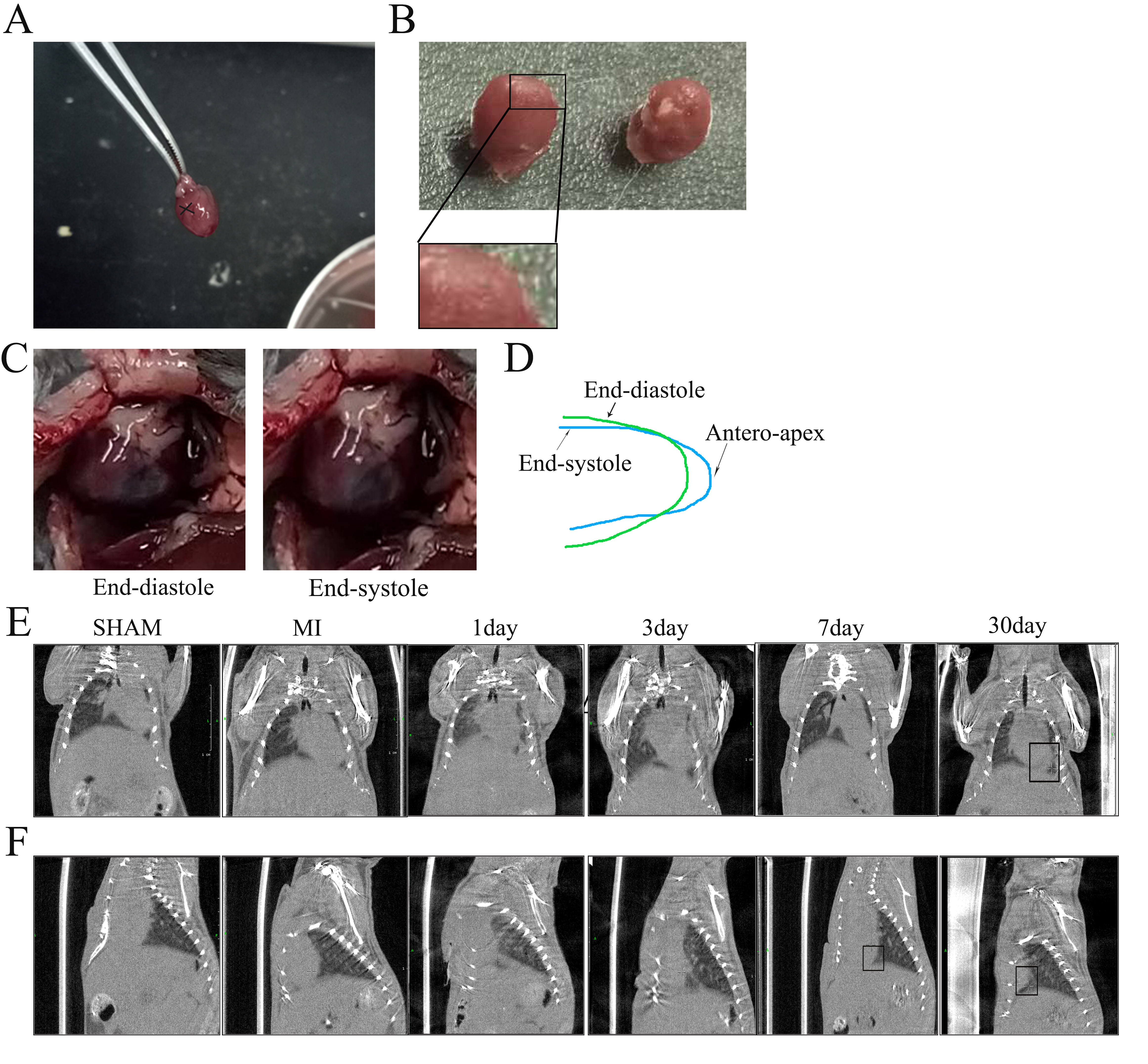

Supplement: Supplementary Figure 1 — A simple, consistent, and low-cost LVA mice model construction. (A) Ligation site of heart organ in left coronary artery descending branch. (B) Intra-chamber cast and optical image of LVA (left) and MI (right), illustrating an outward bulge in the free wall of the left ventricle. (C) Optical LVA formation of mice heart: end-diastole (left) and end-systole (right) ligated at 4-4.5mm from its coronary origin. (D) Pattern picture of heart movement in LVA formation. (E, F) Coronal (E) and sagittal (F) position of computed tomography of LVA formation. SHAM and MI were 7d capture after model construction (n=6 per group). LVA formation was labeled with rectangle. [file Image_1.tif]

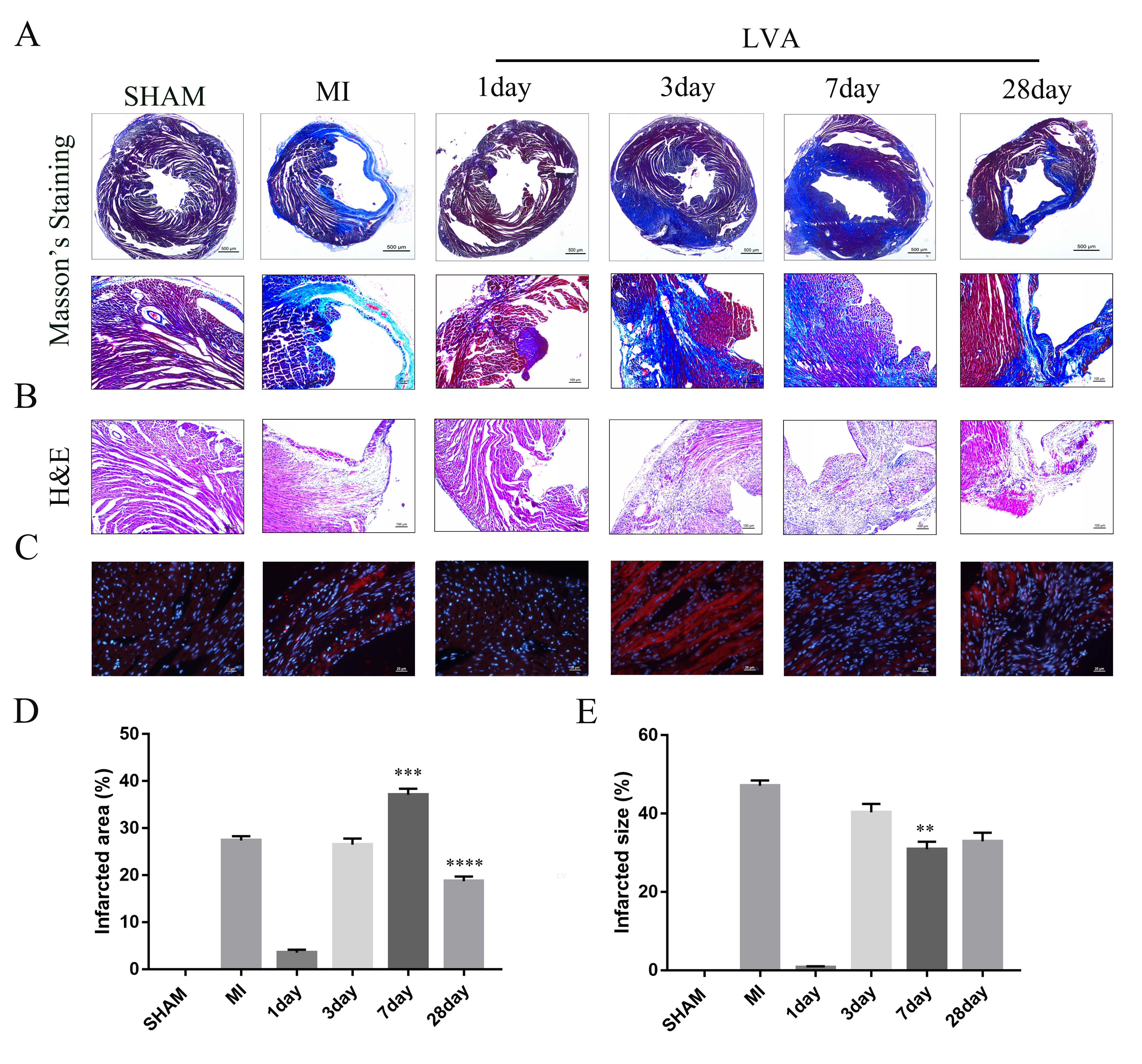

Supplement: Supplementary Figure 2 — LVA formation process. (A) Masson’s staining of representative images showed the LVA forming process in the total scenery (up, 25x) and the border regions (down, 100x) at 1d, 3d, 7d and 28d after operation. The SHAM and MI images at 7d after operation were as negative and positive control, respectively. Scale bar, 500μm and 100μm. (B) HE staining at border zone of representative images at the same d. Scale bar, 100μm. (C) Representative images of heart sections at border zone stained for cTNT (red) at the same day. Scale bar, 25μm. (D–F) Comparison of infarction area (D) and infarction size (E) in border zone compared to SHAM group. ** P<0.01, *** P<0.001, **** P<0.0001. [file Image_2.tif]

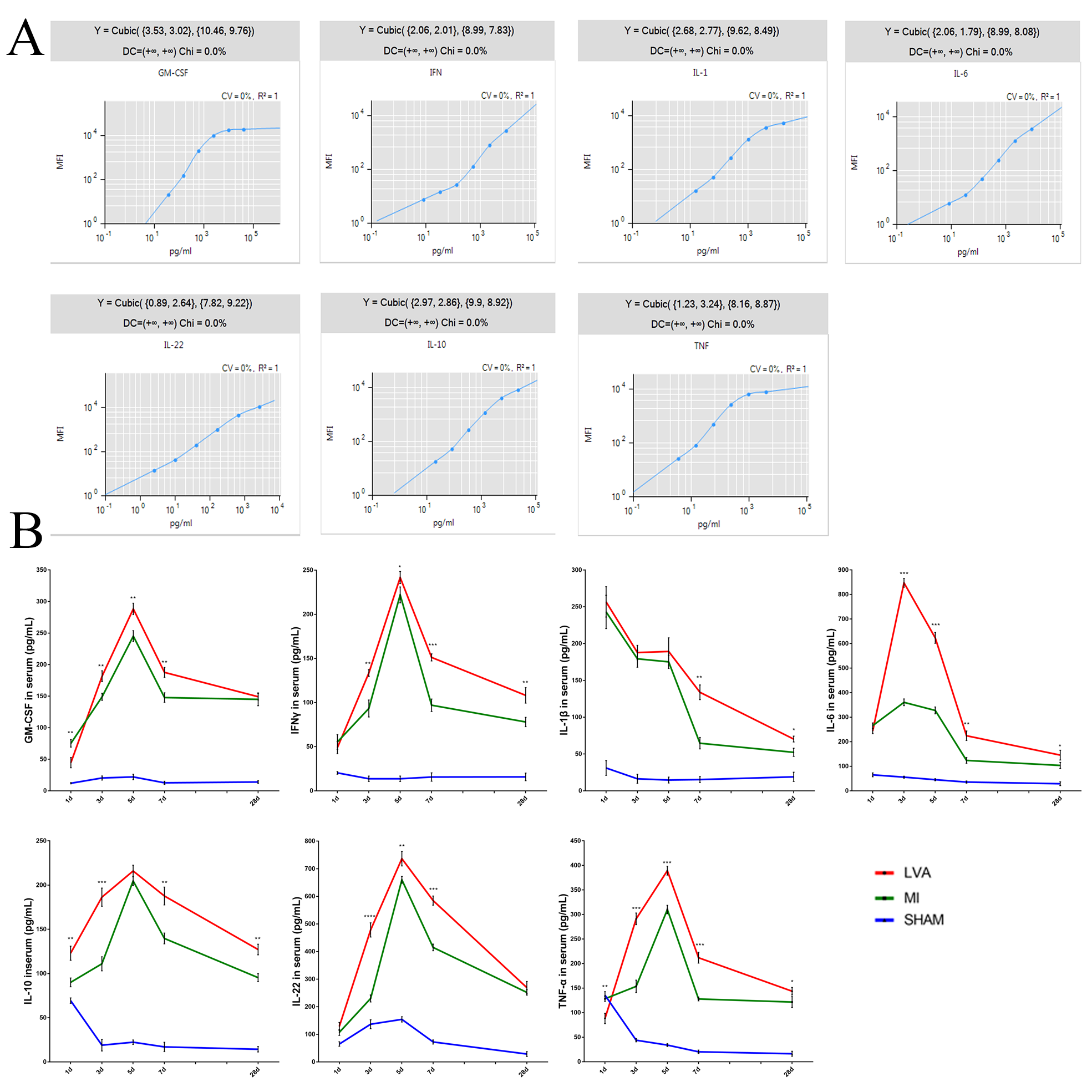

Supplement: Supplementary Figure 3 — Activation of pro-inflammatory and anti-inflammatory during LVA formation and progression. (A) The standard curve of the cytokine and chemokine measurements: IFN-ur GM-CSF, IL-1SF IL-6, IL-10, IL-22 and TNF22 (B) The concentrations of the cytokine and chemokine measurements: GM-CSF, IFNCS IL-1SF IL-6, IL-10, IL-22 and TNF2 among the three groups at 1d, 3d, 5d, 7d and 28d after operation (n=5 per group). LVA vs. MI: * P<0.05; ** P<0.01; *** P<0.001; **** P<0.0001. [file Image_3.tif]

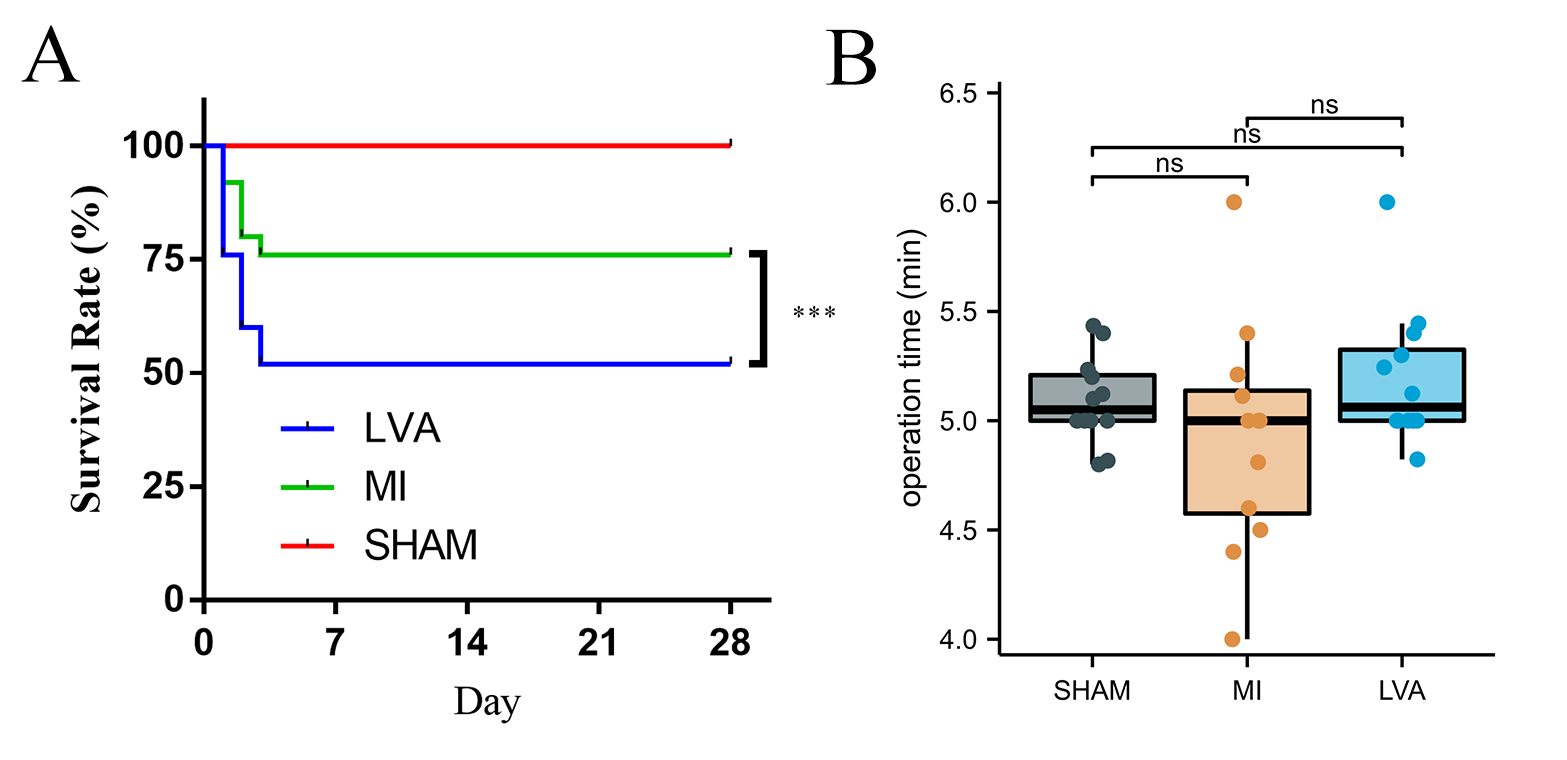

Supplement: Supplementary Figure 4 — The survival rate among 3 groups. (A) The survival rate of LVA, MI and SHAM (n=25, 25 and 20, respectively). (B) Operation time of LVA, MI and SHAM (n=12 per group). [file Image_4.tif]

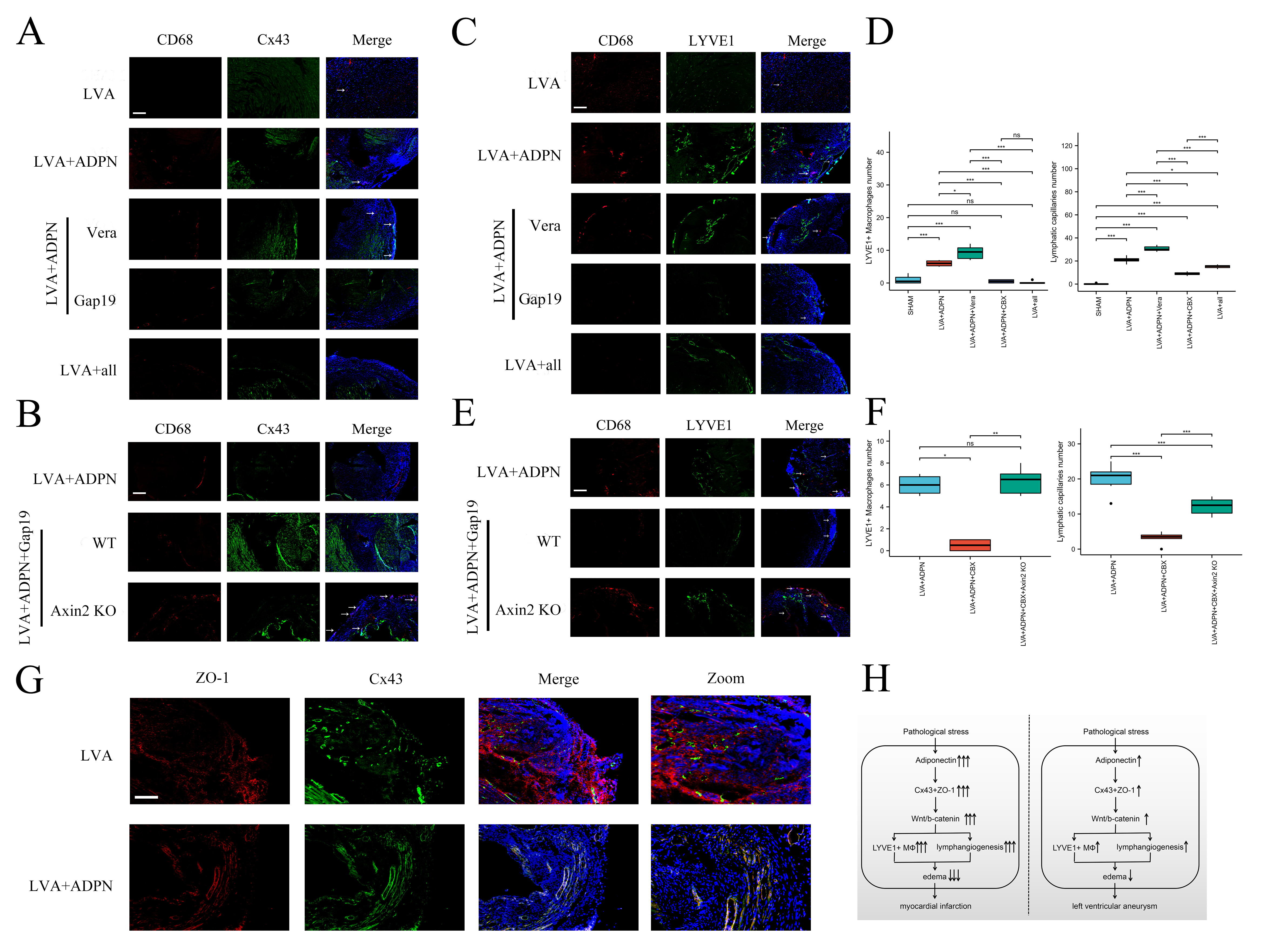

Supplement: Supplementary Figure 5 — ADPN regulated LYVE1+ macrophages and lymph-angiogenesis via Cx43 and Wnt canonical signaling. (A) The representative immunofluorescence images of Cx43 expression and Cx43 associated macrophages in the LVA mice treated with ADPN, verapamil and/or Gap19 from day 1 to 7. (B) The representative immunofluorescence images of Cx43 expression and Cx43 associated macrophages in WT or Axin2 KO LVA mice treated with ADPN and Gap19 from day 1 to 7, demonstrating that consistent activation of Wnt canonical signaling rescued the effects of downregulated Cx43 expression on Cx43 associated macrophages infiltration. (C, D) The representative immunofluorescence images (C) and quantity analysis (D) of LYVE1+ macrophages and lymph-angiogenesis in the LVA mice treated with ADPN, verapamil and/or Gap19 from day 1 to 7. (E, F) The representative immunofluorescence images (E) and quantity analysis (F) of LYVE1+ macrophages and lymph-angiogenesis in WT or Axin2 KO LVA mice treated with ADPN and Gap19 from day 1 to 7, demonstrating that consistent activation of Wnt canonical signaling rescued the effects of downregulated Cx43 expression. (G) The representative immunofluorescence images of Cx43 and ZO-1 expression and localization in the LVA mice treated with ADPN or control from day 1 to 7. (H) The mechanism diagram of MI and LVA formation. The ADPN expression level differed after coronary occlusion, leading to the significant difference of lymph-angiogenesis number and infiltrated LYVE1+ macrophages, thus inducing different mice model, such as MI and LVA formation. Scale bar, 500μm. * P<0.05; ** P<0.01; *** P<0.001; ns, not significant. Arrow, LYVE1+ macrophages. Scale bar, 500μm. [file Image_5.tif]

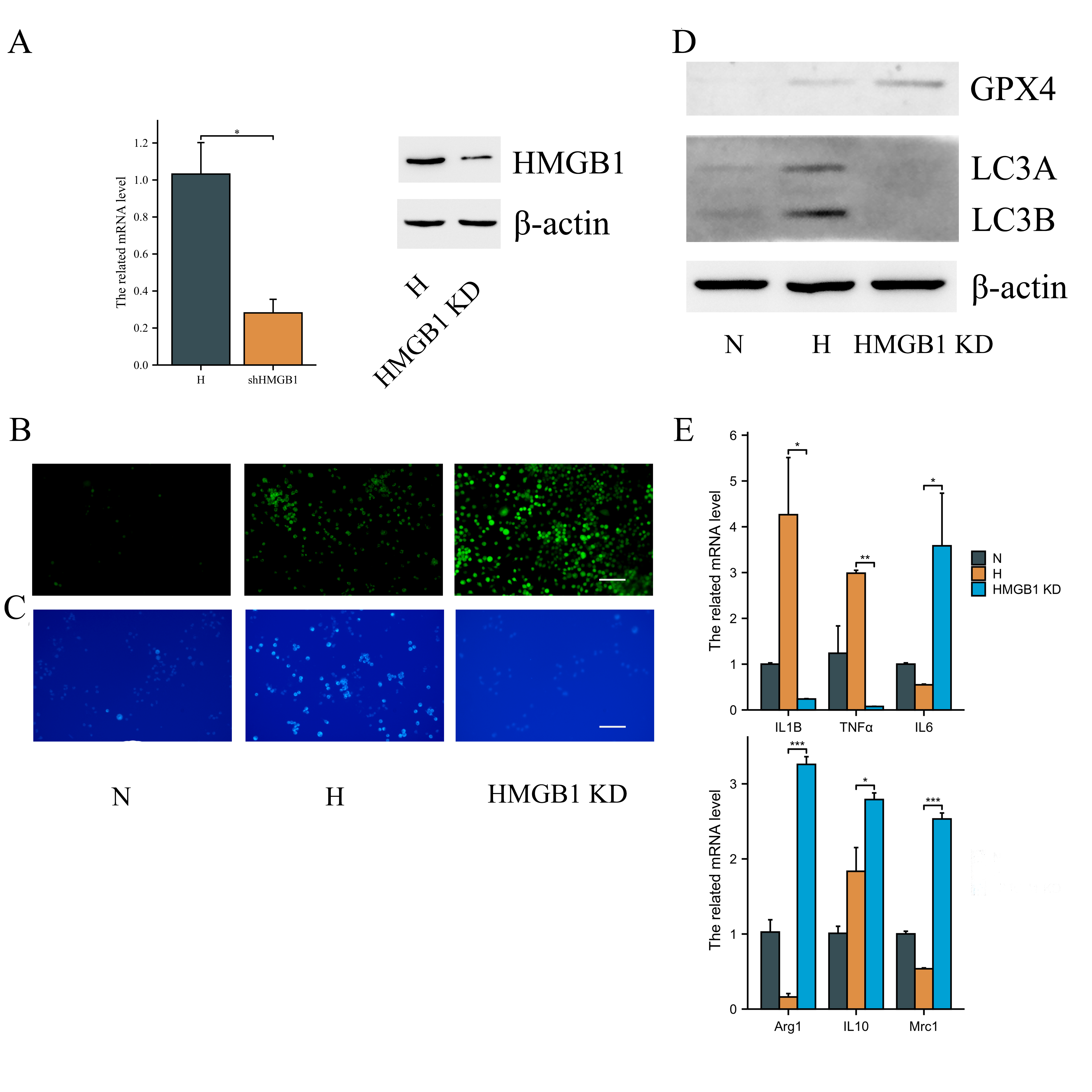

Supplement: Supplementary Figure 6 — HMGB1 knockdown led to mitochondrial quality control, autophagy reduction and M2b polarization in hypoxic macrophages. (A) qPCR and WB analysis demonstrated the HMGB1 knockdown efficiency in hypoxic macrophages. (B) ROS measurement in normal oxygen, hypoxia and HMGB1 knockdown Raw264.7. (C) Autophagy measurement in normal oxygen, hypoxia and HMGB1 knockdown Raw264.7 using MDC method. (D) The represented WB images of GPX4 and LC3 protein expression in normal oxygen, hypoxia and HMGB1 knockdown Raw264.7. (E) The related mRNA levels of IL1b, TNFa, IL6, Arg1, IL10 and Mrc1 in N, H and HMGB1 KD group. KD, knockdown; N, normal oxygen Raw264.7; H, hypoxia Raw264.7; HMGB1 KD, hypoxia HMGB1 knockdown Raw264.7. Scale bar, 100μm. *P<0.05; **P<0.01; ***P<0.001. [file Image_6.tif]

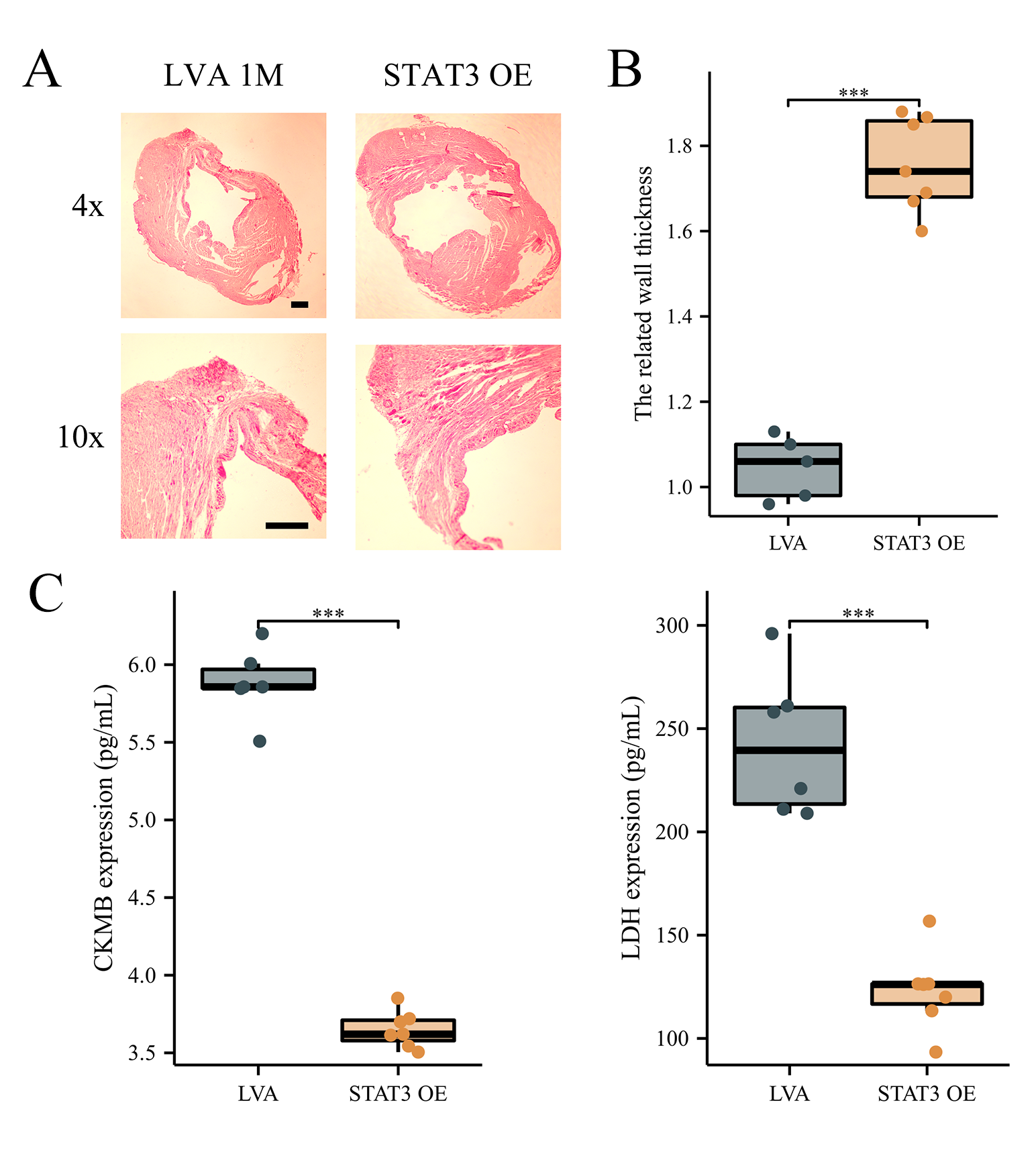

Supplement: Supplementary Figure 7 — Supplying STAT3 overexpressed adipocytes decreased injury after LVA formation. (A) The HE representative images of heart sections in LVA and LVA+STAT3 overexpressed adipocytes groups. (B) The related wall thickness of border zone in LVA and LVA+STAT3 overexpressed adipocytes groups. (C) The border zone CKMB expression and LDH expression at 7d after operation in LVA and LVA+STAT3 overexpressed adipocytes groups using ELISA. Scale bar, 500μm. ***P<0.001. [file Image_7.tif]

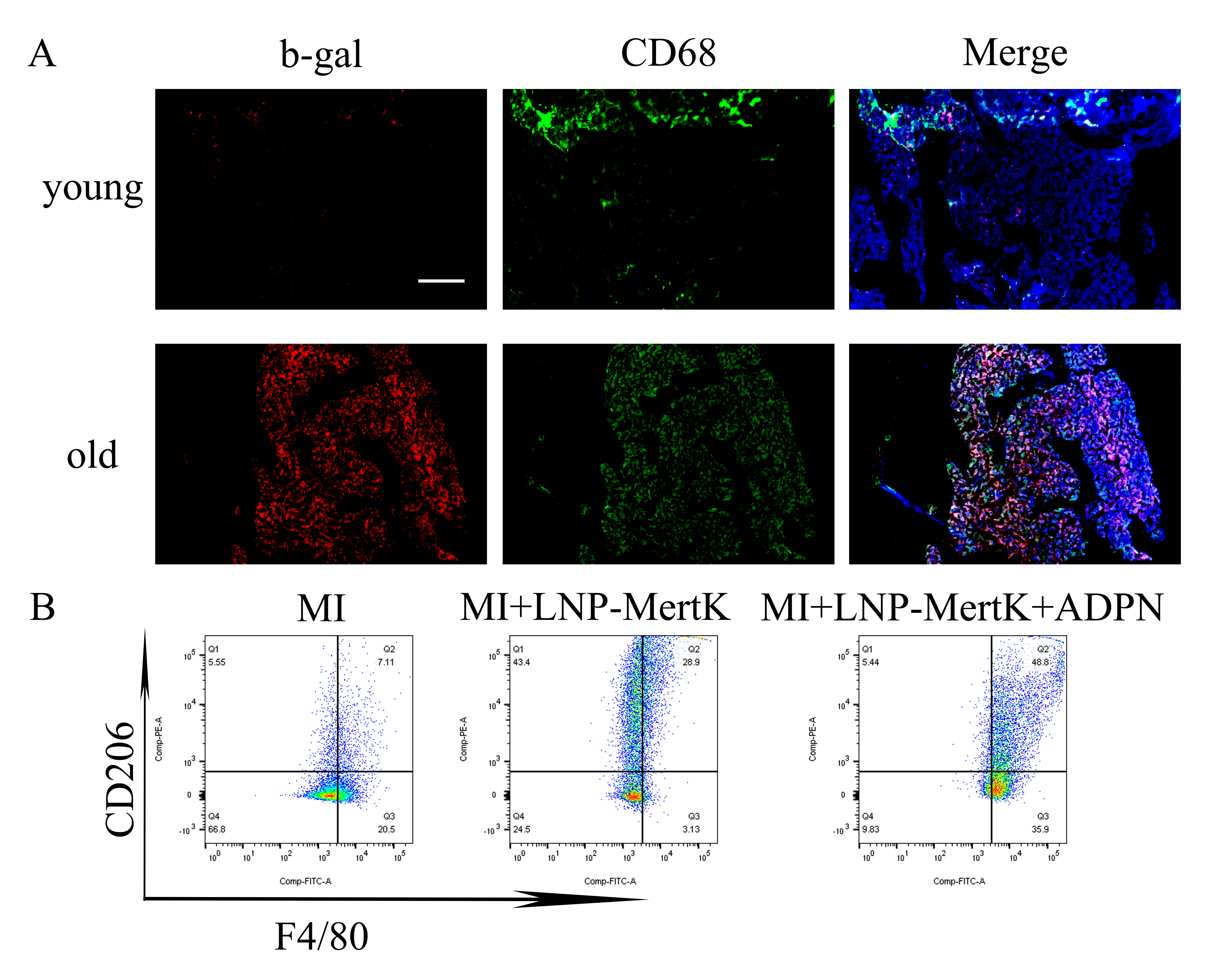

Supplement: Supplementary Figure 8 — Aged macrophages hided in bone marrow. (A) The immunofluorescent images with b-gal and CD68 in bone marrow in young and aged MI mice. (B) Flow cytometry of F4/80 and CD206 staining to determine the macrophage M2 polarization in border zone of aged MI mice when treated LNP-MertK or LNP-MertK+ADPN at 7d after operation. [file Image_8.tif]

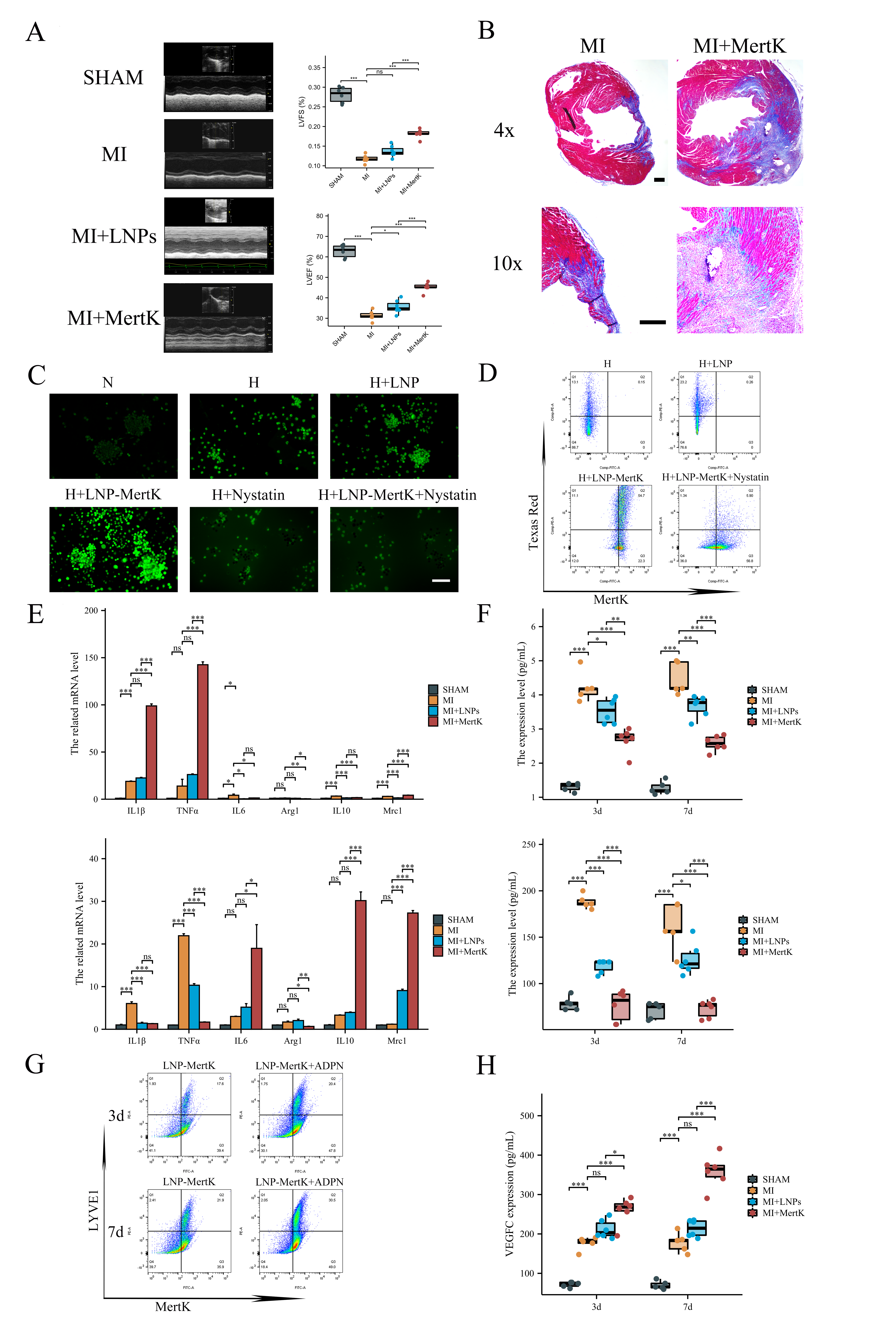

Supplement: Supplementary Figure 9 — Targeting MertK improved the heart functions after MI. (A) The representative echocardiogram image and quantity analysis, including LVEF and LVFS, in the SHAM, MI, MI+LNP and MI+LNP-MertK groups. (B) The representative images of Masson’s staining in the MI and MI+LNP-MertK groups. Scale bar, 500um. (C) HL-1 cardiomyocytes were pretreated with JC-1 and hypoxia 4h. Green labeled represented bad mitochondrial function. After that, transwell analysis was applied to cardiomyocytes (up) and macrophages, LNP treated macrophages or LNP-MertK treated macrophages (down). Nystatin was utilized to rescue the effects of LNP-MertK. Scale bar, 100um. (D) MitoTracker® Red was used to pretreat hypoxic cardiomyocytes and transwell analysis and flow cytometry were used to analyze the protective effects of LNP-MertK on myocardial mitochondrial. (E) The macrophage polarization in four groups at 3d (up) and 7d (down) after operation. (F) The CKMB expression (up) and LDH expression (down) at 3d and 7d after MI using ELISA. (G) Flow cytometry of LYVE1 and MertK staining to determine the abundance of infiltrated phagocytic macrophages when treated with LNP-MertK or LNP-MertK+ADPN. (H) VEGFC expression was determined using ELISA at 3d and 7d after operation in the SHAM, MI, MI+LNP and MI+LNP-MertK groups. * P<0.05; **P<0.01; ***P<0.001; ns, not significant. [file Image_9.tif]
